# Clinical Effectiveness of Dry Needling in Patients with Musculoskeletal Pain—An Umbrella Review

**DOI:** 10.3390/jcm12031205

**Published:** 2023-02-02

**Authors:** Marjolein Chys, Kayleigh De Meulemeester, Indra De Greef, Carlos Murillo, Wouter Kindt, Yassir Kouzouz, Bavo Lescroart, Barbara Cagnie

**Affiliations:** 1 Department of Rehabilitation Sciences, Ghent University, 9000 Ghent, Belgium; 2 Pain in Motion International Research Group, 1000 Brussels, Belgium

**Keywords:** dry needling, umbrella review, pain, disability, physical functioning, trigger point, musculoskeletal disorder

## Abstract

The number of systematic reviews (SR) summarizing the literature regarding the clinical effects of Dry Needling (DN) has increased rapidly. Yet, rigorous evidence about the clinical effectiveness of this technique is still lacking. The aim of this umbrella review is to summarize the evidence about the clinical effects of trigger point DN on musculoskeletal disorders across all body regions. PubMed, Web of Science and Embase were searched to identify SRs examining the effect of DN (as a stand-alone intervention or combined with another treatment modality) compared to sham/no intervention or a physical therapy (PT) intervention with at least one clinical outcome in the domain of pain or physical functioning. Risk of bias (RoB) was assessed with the AMSTAR-2 tool. Quantification of the overlap in primary studies was calculated using the corrected covered area (CCA). The electronic search yielded 2286 results, of which 36 SRs were included in this review. Overall, DN is superior to sham/no intervention and equally effective to other interventions for pain reduction at short-term regardless of the body region. Some SRs favored wet needling (WN) over DN for short-term pain reductions. Results on physical functioning outcomes were contradictory across body regions. Limited data is available for mid- and long-term effects. DN has a short-term analgesic effect in all body regions and may be of additional value to the interventions that are used to date in clinical practice. Several studies have shown an additional treatment effect when combining DN to physiotherapeutic interventions compared to these interventions in isolation. There is a substantial need for the standardization of DN protocols to address the problem of heterogeneity and to strengthen the current evidence.

## 1. Introduction

Musculoskeletal (MSK) pain disorders are prevalent in the general population and are associated with long-term pain and disability [1,2,3]. In the last two decades, dry needling (DN) has become extremely popular in the management of MSK pain and related disability, either as a stand-alone treatment or in combination with other therapeutic interventions. Dry needling is a treatment modality that is minimally invasive, cost-effective, easy to learn with appropriate training, and carries a low risk of complications [4]. Yet, the effectiveness of this novel treatment technique is still under discussion and results vary widely over the published literature [5].

The most common and best supported DN approach targets myofascial trigger points (MTrPs) and aims to induce peripheral and central physiological and clinical effects [5,6,7,8]. Among patients with acute and chronic MSK disorders, myofascial pain syndrome is often present and is described as one of the underlying causes of the dysfunction [9,10,11]. It is characterized by sensory, motor, and autonomic symptoms caused by MTrPs. Patients usually present with localized pain in a restricted area or various referred pain patterns. Several clinical effects have been described for DN, of which the analgesic effect is the most reported outcome. Other suggested clinical effects include improvements in physical functioning, such as changes in disability, range of motion or muscle strength [5,6,8].

Over the past years, the number of systematic reviews (SRs) with or without meta-analyses (MAs) summarizing the literature regarding the clinical effects of DN has increased rapidly. Systematic reviews are at the top of the evidence pyramid, and healthcare decision makers rely on systematic reviews as one of the key tools for achieving evidence-based healthcare [12]. Nevertheless, considering the growing amount of evidence across the multiple body regions, it is difficult to make well-informed choices. Therefore, an umbrella review (UR) allows the comparison of findings of several SRs, considering for inclusion only the highest level of evidence [13].

Consequently, this UR aims to synthetize all published SRs on trigger point DN and its clinical effectiveness in patients with myofascial pain across several MSK conditions.

## 2. Materials and Methods

This UR was registered in PROSPERO (CRD42022330512) and followed the Preferred Reporting Items for Systematic Reviews and Meta-Analyses PRISMA-2020 guidelines and the Joanna Briggs Institute methodology for URs [13,14].

### 2.1. Eligibility Criteria

Systematic reviews with or without meta-analyses were eligible if they met the following criteria: inclusion of (P) participants with acute to chronic MSK pain disorders, age 18–65 years; (I) receiving at least one DN session (as a stand-alone intervention or combined with another treatment modality [e.g., therapeutic exercise]); (C) compared to sham/placebo, no intervention or other (active or passive) interventions (e.g., manual therapy (MT), exercise therapy, WN etc.); (O) with at least one clinical outcome in the domain of pain or physical functioning(e.g., active and passive range of motion, strength, functionality, disability, quality of life or daily life activity) [15,16]. (S) Included articles were SRs of only RCTs, with or without MA, written in Dutch or English (published since the year 2000). More information about the eligibility criteria can be found in Appendix A.

### 2.2. Information Sources and Search Strategy

The databases PubMed, Web of Science and Embase were searched up to 2 June 2022 (Supplementary Materials: Tables S1 and S2). Additionally, reference lists of the included SRs and trial registries were hand-searched to identify additional studies not identified through electronic searches. No language or publication year restrictions were applied.

### 2.3. Selection Process

The studies identified through the database and hand search were assessed for eligibility by two independent reviewers using a 2-stage process. First, all identified records were screened based on title and abstract. Secondly, the full text of the remaining articles was assessed for eligibility. Any disagreements were resolved through discussion at each stage, and, if consensus was not reached, an additional reviewer was consulted.

### 2.4. Data Collection Process

The data was extracted from the articles by two independent reviewers and checked by a third reviewer. The extracted data included (i) author, publication year, country and SR design (SR and/or MA); (ii) information on the study sample (including number and publication years of RCTs, and number of patients per treatment arm); (iii) treated body region(s); (iv) details of the interventions (i.e., DN and comparator); (v) outcome(s) (i.e., measurement tool and follow-up); (vi) results; (vii) adverse events and (viii) remarks. If the follow-up period was not specified in the SR, the range of follow-up periods used in the included RCTs was provided [17].

### 2.5. Study Risk of Bias Assessment

The quality of the SRs was assessed by three independent reviewers using the AMSTAR 2 checklist for systematic reviews [18]. Any discrepancies were resolved through discussion and consensus. Inter-rater agreement of the AMSTAR 2 assessment was calculated using the Cohen’s Kappa coefficient ($\kappa =\frac{p_{0}-p_{e}}{1-p_{e}}$). Additionally, the overall assessment as suggested by Shea et al., (2017) was implemented to generate an overall score of the quality of the included SRs (high, moderate, low, and critically low). Items 4, 9, 11 and 13 were considered to be critical flaws.

### 2.6. Synthesis of the Results & Data Analysis

The degree of overlap in primary studies in the included SRs was calculated using the corrected covered area (CCA) and generating a citation matrix [19,20]. To characterize overlap by needling area, CCA calculations for pairs of reviews were performed and presented as a grid. Overlap thresholds were used for the interpretation of measured overlap (0–5%—slight, 6–10%—moderate, 11–15%—high, >15%—very high) [19]. The results were selected from a subset of SRs according to a prespecified decision rule and published algorithm [21]. In case of very high overlap, the authors selected the SR with the highest quality. If the methodological quality was the same, the most recent SR was selected. When two or more SRs reported data for the same outcome and were published within the same year, the review including the greatest number of primary studies was selected [19,21,22,23,24,25].

A strong recommendation was made when at least 50% of the SRs considering a specific topic had at least moderate-level evidence, with at least one review having high-level evidence. A moderate recommendation was made when at least 50% of the reviews had moderate-level evidence. A weak recommendation was made when fewer than 50% of the reviews had moderate-level evidence [26]. Recommendations were only made if at least three reviews of low to high methodological quality were available for a specific body region. Reviews categorized as critically low were excluded from the synthesis of the results, since it was judged that the review outcome would not provide an accurate summary of the available evidence [27].

## 3. Results

### 3.1. Study Selection

**Figure 1:**
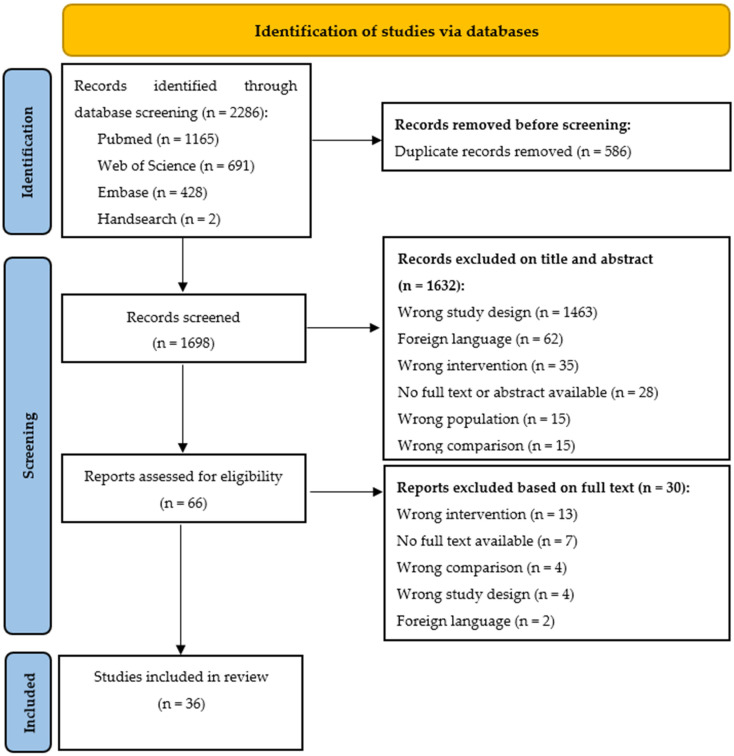
Prisma flow diagram.

The database search resulted in 2286 SRs, of which 1699 SRs remained after duplicate removal. After the first screening of titles and abstracts, 66 SRs were retrieved for full text screening. Finally, 36 SR (12 SRs without and 24 SRs with MA) were included in the final synthesis. Further details on the screening process can be found in the flow diagram (Figure 1). Combined, the SRs included 210 primary studies, with a total N = 24869 (See Supplementary Materials: Table S3: Reference list of primary studies). The total amount of overlap was slight (3.28%) for all included articles (total CCA); however, the CCA for the total body, UQ and LQ articles was slight to moderate, with percentages of 7.06%, 2.54% and 7.14% respectively. The amount of overlap is presented in the Supplementary Materials (Tables S4–S6: Overlap for pairs of reviews; Table S7: Citation Matrix).

### 3.2. Characteristics of Included Systematic Reviews

Eleven SRs examined DN over the whole body. Fifteen SRs focused on the upper quarter (i.e., seven on the neck, three on the neck and shoulder, three on the shoulder, three on the temporomandibular joint region (TMJ), and one on the elbow). Eight SRs focused on the lower quarter (i.e., two on a general overview of the lower extremity, two on low back pain, two on the knee and two on the heel). All SRs included participants with acute (<3 months) to chronic (>3 months) MSK complaints.

Eleven SRs evaluated the effect of DN as a stand-alone intervention; only one SR evaluated the effect of DN + other interventions. Most of the SRs (67%) included a combination of RCTs investigating DN alone and DN + other interventions; of which only a subset of SRs made a clear distinction in the results between DN as a stand-alone treatment or DN + other interventions. There was a very large heterogeneity in the number of treatments in the included SRs.

Almost all SRs (94%) included a combination of different comparators: sham/placebo interventions (31 SRs), TAU/waiting list/no intervention (9 SRs), or other interventions (35 SRs) such as MT, PT, WN, exercise therapy, stretching, medication, TENS, PENS, ultrasound, etc.). Only 36% (13/36) of the SRs divided the results into two different comparator categories (i.e., sham/placebo/no intervention vs. other interventions. There was no information on primary or secondary outcomes when comparing DN with waiting lists/treatment as usual or with normal care.

The primary outcome measure was pain intensity, included in 35 SRs (198 RCTs in total; *n* = 23892) and was most measured by the VAS/NPRS. Additionally, outcomes related to physical functioning were included in 29 SRs (117 RCTs in total, *n* = 17854). The most reported secondary outcome measure was disability (21 SRs, 229 RCTs in total; *n* = 13341), followed by range of motion (18 SRs, 248 RCTs in total; *n* = 11475). Only 4 (11%) SRs used strength as an outcome measure [28,29,30,31]. A summary of all details and characteristics of the included SRs is presented in Appendix A.

### 3.3. Quality Assessment

The results from the AMSTAR quality appraisal are presented in Table 1. Two studies obtained a high overall score, 19 scored moderate, 11 scored low and 4 were critically low. Most common limitations for SRs were (a) not establishing the methods prior to the conduct of the review [10/36]; (b) a missing explanation of the selection of the study designs for inclusion in the review [28/36]; (c) not providing a list of excluded studies and justification of the exclusions [23/36]; (d) not reporting on the sources of funding for the studies included in the review [34/36]; and (e) a missing explanation of heterogeneity in the results [23/36] [18]. There was a substantial agreement (89%) between authors in the rating of the quality of the SRs (k = 0.74).

### 3.4. Synthesis of Results

#### 3.4.1. Whole Body

Eleven SRs, including 102 unique RCTs, examined the effects of DN on several body regions [28,31,32,33,34,35,36,37,38,39,40]. Due to very high overlap (see Appendix A: pairs of reviews) and the very poor methodological quality of some of the included articles, nine SRs [28,31,32,33,34,35,36,38,39] were considered when summarizing these results. Recommendations for whole body and upper quarter regions are summarized in Table 2.

- Pain intensity

DN compared to sham/placebo DN was shown to be superior for short-term pain reduction [31,32,34,35,36,38]. There is low-quality evidence suggesting a moderate effect favoring DN over control/sham immediately to 12-weeks post-intervention. There is moderate-quality evidence suggesting a small effect favoring DN over control/sham in the long-term (6–12 months follow-up) [35].

DN compared to other interventions: Two MAs found moderate quality evidence suggesting small [35] to moderate [34] effects favoring DN over other interventions in the short-term (immediate to 12-weeks) [35]. DN was at least equally effective as manual MTrP release and other needling treatments [34]. DN in combination with other therapies was more effective than applying the other therapies alone at short-term [38]. One large SR (42 RCTs, N = 3967) found low quality evidence for a large effect of DN (with or without other treatments) compared to other therapies immediately, at mid- and at long-term for reducing pain [38]. A decrease in pain intensity at long-term (13–24 weeks) was found when analyzing DN against other therapies or when comparing DN + other therapies with these therapies alone. Two SRs found similar results for DN compared to WN [36,39]. Cummings et al. found that DN and WN are equally effective for pain intensity reduction, and the follow-up was not specified [33]. CSI (corticosteroid injection) seems to be more effective than DN in the short-term for the management of heel and lateral elbow pain, yet DN seems to be more effective at long-term follow up [39]. The effects of CSI and DN were similar for myofascial and greater trochanteric pain regardless of the follow-up period [39].

- Physical functioning

DN compared to sham/placebo: There is low-quality evidence suggesting a small effect favoring DN over control/sham for changes in functional outcomes at short (immediately—12 weeks) and long-term (6–12 months) follow-up [35]. DN is effective for improving quality of life and range of motion in the neck and shoulder compared to sham/placebo at short-term [34]. Only a few studies [31,34,36] evaluated changes in range of motion, and the results were contradictory. There was insufficient evidence to support the use of DN as an intervention to increase strength, except for the cervical spine (moderate strength of evidence) [28]. There was insufficient information on long-term effects [28].

DN compared to other interventions: DN is equally as effective as other interventions (MT, WN or pharmacological interventions) for improvements in range of motion, disability and quality of life [32,34]. One SR found very low-quality evidence suggesting a no treatment effect for changes in functional outcomes compared to other treatments at short-term [35].

#### 3.4.2. Upper Quarter

##### Temporomandibular Dysfunctions

Three SRs discussing DN in the temporomandibular joint region (TMJ) were included [41,48,55]. The results should be interpreted with caution due to the very high amount of overlap between SRs and MAs.

DN compared to sham/placebo: There was very low-quality evidence that no statistically significant difference was found between DN and sham for short-term orofacial pain [55]. There was no between-group difference in short-term pain-free maximal mouth opening (MMO) (very low-quality evidence) [55]. This contrasts with Al Moraissi et al., who found a significant improvement in MMO after DN versus placebo [41].

DN compared to other interventions: In terms of pain intensity, no significant differences were found in the short-term between DN or WN. Two reviews found a significant effect favoring DN compared to other treatments (medication, laser therapy, stretching) [48,55]. Al-Moraissi et al. described the top three highest ranked treatments for pain reduction at short (1–20 days) and mid-term (1–6 months), with DN being part of the top three in both rankings. They also found local anesthesia to be the most effective treatment regarding the increase in ROM (maximal mouth opening), followed by DN.

##### Headache

One high quality SR with MA showed that DN could significantly improve headache frequency, health-related quality of life, trigger point tenderness, and cervical ROM in a tension type headache (TTH) and cervicogenic headache (CGH). DN produced similar effects to other interventions for short-term headache pain relief but seemed better than other therapies for improvement in related disability in the short-term [53].

##### Neck

This UR included nine SRs that studied DN in the neck [43,44,45,46,47,49,51,52,54]. Based on the amount of overlap and the methodological quality of the reviews, six studies were withheld to write these results [43,44,45,46,49,51].

- Pain intensity

DN compared to sham/placebo: DN produced an analgesic effect immediately after treatment and at short-term [45,49].

DN compared to other interventions: DN was not superior to other interventions [43,44,45,46,49,51]. There was low quality evidence that lidocaine injection exhibits a superior effect for reducing pain compared to DN at short-term [45,51]. A limited number of studies evaluated the mid-and long-term effects [43,49,51]. One recent high-quality MA examined the short- to long-term effects of the added value of DN to another intervention (MT, PT etc.) compared to the intervention alone or DN alone [44]. The combined interventions showed significantly larger effects for reducing pain intensity as compared to the interventions in isolation in the short-term. At mid-term, there was a significant small effect and at long-term, no significant effect on pain intensity was observed.

- Physical functioning

DN compared to sham/placebo: Two SRs found significant effects of DN over sham/placebo interventions [43,49].

DN compared to other interventions: For reducing disability or improving functionality, most reviews found comparable results of DN compared to other interventions (MT, WN, other PT interventions) in the short and mid-term [43,46,49,51]. DN can be of added value in improving disability in the short-term, since effects of the combined interventions were better than the interventions as a stand-alone treatment [44]. Four SRs evaluated changes in range of motion [43,44,49,51]. Three did not observe significant differences between groups at any time point compared to WN [51], ischemic compression/lidocaine injection [43] or either comparative intervention [49]. One MA observed a significantly small short-term effect of DN combined with other interventions against other interventions alone on cervical ROM in all directions [44].

##### Shoulder

- Pain intensity

DN compared to sham/placebo: Two MAs found statistically significant effects of DN compared to sham at short-term; these results were confirmed in the mid-term but not at long-term evaluation [47,50].

DN compared to other interventions: Moderate to low-quality evidence suggests that the positive (small) effects of DN in non-traumatic shoulder pain of MSK origin at short-term [50]. Mid-term results were in favor of WN or other treatments compared to DN. One SR found significant. improvements in pain intensity for patients with subacromial syndrome [42]. Changes were not maintained during the follow-up period and comparison groups were not adequately defined [42,50].

- Physical functioning

Compared to other interventions: There was low quality evidence that DN improves disability with a large effect in non-traumatic shoulder pain [50]. For changes in functionality and disability, DN was better than [50], or equally effective to other interventions [42,50], but only at short-term. However, two RCTs found a statistically significant improvement in functionality when DN was added to a standard PT regime. One SR with heterogeneous comparison groups studied ROM. Results varied widely between studies and no firm conclusions about ROM can be drawn [42].

##### Elbow

Compared to other interventions: One SR studied the clinical effects of DN patients with lateral epicondylalgia [30]. DN reduced pain intensity and related-disability with large effect sizes compared to a heterogeneous comparative group at short- and long-term, but not immediately. There was also an increase in grip strength (small size effect) at short-term.

#### 3.4.3. Lower Quarter

Khan et al. [58] and Morihisa et al. [61] included 16 RCTs comparing DN in the lower quadrant. Both reviews concluded that DN was an effective intervention for reducing pain associated with lower quarter MTrPs at short-term. RCTs comparing DN with sham or placebo showed marked improvements in pain; when comparing DN with other therapeutic modalities or MT, the results had similar results for pain reduction [58]. There was inconclusive evidence for positive short- or long-term effects on changes in physical functioning (quality of life, range of motion or strength). Combining DN interventions with other therapeutic interventions (e.g., stretching and exercise) was demonstrated to have an additional advantage [61].

##### Low back

This UR included two studies with a very high percentage of overlap (CCA = 68.75%) [57,59].

- Pain intensity

Compared to sham/placebo: Both reviews found DN to be superior for pain intensity immediately post-intervention when compared to SN. The effects were maintained at follow-up (not specified) [57].

Compared to other interventions: Both reviews found DN to be superior immediately post-intervention when compared to acupuncture. The effects were not maintained at follow-up. When compared to other interventions (apart from acupuncture), the results were inconsistent and strongly dependent on the type of intervention and dosage of treatment [57]. Hu et al. described the results quantitatively, while Liu et al. performed an MA and found superior results for DN over other interventions at short-term. The results were not maintained at follow-up. Liu et al. compared the effect of DN alone vs. DN plus other treatments and found evidence favoring DN plus other treatments for pain reduction at post-intervention but not for improvement in disability [59].

- Physical functioning

Compared to sham/placebo: Both reviews found DN to be superior for disability immediately post-intervention when compared to SN. At follow-up, no significant differences were found.

Compared to other interventions: Both reviews found DN to be superior for disability immediately post-intervention when compared to acupuncture. At follow-up, no statistically significant differences were found.

##### Knee

Only 2 studies on knee pain were found [62,63]. Due to the amount of overlap (11.76%), the (very low) methodological quality and the limited number of studies about DN in the review of Ughreja et al., only one MA [62] will be discussed.

Rahou-El-Bachiri et al. found a significant moderate effect size for decreasing pain intensity and disability in the short-term. They found no significant differences between DN and a comparative group (mix of comparators: sham/placebo/other interventions) in the mid- and long-term. There is low to moderate evidence supporting a positive effect of DN on pain and disability in patellofemoral pain, but not in osteoarthritis or post-surgical knee pain at short-term [62].

##### Heel

Two studies including 13 RCTs (with high overlap; CCA = 18.18%) examined the effectiveness of DN for plantar heel pain or plantar fasciitis [56,60].

Both studies found that DN significantly improves the pain intensity when compared to the comparison group (mix of comparators: sham/other interventions) at short- and long-term. There was low quality evidence that DN reduces pain intensity in the short-term and moderate quality evidence for improving pain intensity and related disability in the long-term, as compared with a comparison group (mix of comparators) [60]. It seemed that fewer than three sessions may not be enough to improve pain in individuals with plantar fasciitis [60].

## 4. Discussion

To the best of our knowledge, this is the first UR evaluating the clinical effects of DN in patients with MSK pain. The current findings, based on the highest methodological quality, suggest that DN is an effective treatment for MTrP-induced pain for short-term pain relief. There is no superiority of DN over other treatments (such as other needling techniques, MT, or exercise/PT) but it may be of additional value to the interventions that are currently used in clinical practice. The current evidence shows that DN is superior to no intervention/sham/placebo for improvements in pain intensity in all body regions. The literature showed conflicting evidence about the comparison of DN to WN. It was suggested that WN might be superior for short-term improvements (up to three months) in pain; however, DN may catch up at mid-term evaluation (3–6 months). Few studies evaluated the mid- and long-term effects with high heterogeneity between trials. In general, there was low quality evidence suggesting a positive effect at mid- and long-term for neck pain, but not for shoulder pain (mid-term results were in favor of WN or other treatments compared to DN). For lateral epicondylitis and plantar fasciitis/heel pain, the results favored DN at the long-term; nevertheless, the conclusions are based on limited data.

There is no conclusive evidence for improvements in range of motion, and results strongly varied across body regions and the included studies. When considering ROM, the results may vary widely due to differences in anatomical regions and in treatment protocols. In addition, numerous studies used DN as a single intervention on one previously determined muscle, which may not be sufficient to exhibit meaningful changes nor reflect a clinical practice setting. When applying DN to muscles with a specific anatomical location and measuring range of motion at this specific location, changes may be present. For example, Murillo et al. targeted a DN intervention to the Obliquus Capitis Inferior muscle and found an immediate and short-term clinically meaningful increase in upper cervical mobility at the C1C2-level compared to SN [64].

Considering improvements in disability or functionality, DN is superior to sham/control/no interventions and equally effective to other interventions. The best option is a combined treatment (conventional physiotherapy with DN), which seems to be more effective than applying the techniques in isolation. Since DN mostly focuses on restoring function by increasing blood flow and diminishing spontaneous electrical activity and disrupting the integrity of a dysfunctional motor endplate [6], its primary effect in acute MSK pain is most efficient in the initial stage when pain, range of motion deficits, and disability are more present. As rehabilitation programs progress, restoring muscle function (motor control), strength and mobility by means of exercise therapy will become more important [65,66]. For chronic pain, several peripheral and central neurophysiological effects have been described as well (such as effects on central sensitization) [6,7,8,67] and needling techniques have recently been added to the treatment guidelines of (chronic) neck pain and low back pain [68,69,70].

No immediate effects on strength, except for neck pain, were established, and the available evidence was conflicting. Nevertheless, since muscle inhibition and motor control deficits may be present during rehabilitation because of acute or chronic pain [71], it may be of interest to evaluate the additional value of DN in a multimodal treatment program, while tracking the changes in motor performance (sensorimotor control and strength) and muscle properties such as excitability, contractibility, extensibility and elasticity.

In almost all included SRs, the terms placebo and sham are used interchangeably. Definitions, methodological descriptions and the evaluation of the sham/placebo effectiveness are lacking. Blinding is widely regarded as crucial to the acceptability of clinical trial outcomes, and trial outcomes are exaggerated when blinding procedures are suboptimal [72]. Considering the complexity of blinding in physical intervention research, two Delphi studies have been performed to evaluate the most important elements of shams for DN research [73]. Experts placed high importance on the entire intervention experience for active and sham protocols. Sham credibility may be maintained using cognitive strategies, potentially relinquishing the need for indistinguishable shams that have proved problematic to design [74]. Furthermore, previous experiences with DN should be taken into account when evaluating the effectiveness of sham procedures [75]. Because there is no widely accepted sham protocol for DN research, researchers should incorporate cognitive influences that extend beyond the mimicking of tactile sensations to create a believable simulation of active dry needling. With regard to the assessment of blinding, using a blinding index might provide more robustness to the results [72,74]. A recent blinding protocol, developed by Braithwaite et al., demonstrated optimal therapist blinding, and near-optimal recipient blinding, making it possible to double-blind dry needling trials (with the caveat that limited needling techniques can be used with the needle devices) [72]. Future trials should consider adequate blinding strategies to learn more about placebo and the real effects of needling interventions.

### 4.1. Heterogeneity of Results and Limitations across Reviews

The most important limitation of almost all included SRs is the high amount of heterogeneity. Next to imprecision, this is one of the most important factors that has led to the downgrading of the evidence as proposed by the GRADE assessment. The most important items for heterogeneity were treatment dosage (number of sessions and frequency of application); the selection of treated muscles, chosen outcome measures, the control group interventions (sham or placebo procedures), follow-up period, needling technique (no explanation or only brief description of the technique, presence of local twitch responses) and the chosen diagnostic criteria for MTrPs. Secondly, the insufficient sample size in certain trials may have led to a publication bias in certain meta-analyses influencing the conclusions at mid- and long-term follow-up.

### 4.2. Strengths and Limitations

This UR used robust methodological approaches based on the most recent published evidence, as described by the Joanna Briggs Institute and including the use of AMSTAR and PRISMA tools [13,14,18]. Nevertheless, an evidence-based and agreement-based reporting guideline for overviews of reviews of healthcare interventions (PRIOR, Preferred Reporting Items for Overviews of Reviews) is still under development [76]. When summarizing the evidence, researchers considered the methodological quality and incorporated methods to deal with overlapping evidence to avoid overweighting the importance of frequently included primary studies. However, there are some remarks to be considered when interpreting our review findings. First, as described above, there was a high heterogeneity among the primary studies. Second, sample sizes varied widely and may have influenced the long-term results. Third, only a limited number of studies discussed the clinical relevance of their results, making the translation from research into clinical practice difficult. Fourth, there were some important methodological considerations; all reviews stated that more high-quality research is necessary with larger sample sizes and interventions, and that blinding techniques should be well described (for example by using the TIDIER checklist), to enhance the reproducibility of the trials.

### 4.3. Clinical Considerations

DN is a safe and effective technique. Minor adverse effects were reported by 47% of the trials, and no major adverse events were present in the 210 unique RCTs, demonstrating that it is a safe intervention when applied by a trained physiotherapist. Since minor complications (small bruising, bleeding, and pain during or after treatment) may be present, clinicians should ensure that the patient is properly informed about the potential risks or side effects. The most reported adverse event was post-needling soreness. Considering the number of treatments necessary for short-term effects, Llurda-Almuzara et al. stated that at least three sessions were necessary for treatment effects [60]. Nevertheless, Espejo-Antúnez et al. found no association between the number of sessions or treatment frequency and pain relief [34]. Sánchez-Infante et al. showed that one session per week proved effective within a 1- to 3-week term [38]. In addition, there is a lack of consistency in the literature on the number of needles that should be inserted and the needle retention time [77,78]. The results are in favor of combined treatments, and DN may enhance treatment efficiency for short-term pain relief. The evidence was conflicting considering the use of WN, nevertheless since the DN technique is easy to apply and more cost-efficient, it may be preferred over WN as a first choice intervention for pain relief.

## 5. Conclusions

There is strong evidence that DN causes pain reduction across all body regions at short-term evaluation. The current evidence shows that DN is superior to no intervention/sham/placebo for improvements in pain intensity. There is no superiority of DN over other treatments, but it may be of additional value to the interventions that are used to date in clinical practice. Several studies have shown an additional effect when combining DN to physiotherapeutic interventions compared to these interventions in isolation. Nevertheless, more research should be done on the possible placebo effects associated this technique. For studies considering LBP and lower quarter MSK pathology, evidence is still more limited. Therefore, no recommendations were made for the LQ. There is a substantial need for standardization of DN protocols to address the problem of heterogeneity and to strengthen the current evidence. Future studies should investigate the mid- and long-term effects of DN.

## Figures and Tables

**Table 1:** AMSTAR Quality Assessment.

Article	1	2	3	4	5	6	7	8	9a	9b	10	11a	11b	12	13	14	15	16	Overall Score
WHOLE BODY																			
Boyles et al. [31]	N	P	Y	Y	Y	Y	N	P	Y	NA	N	NA	NA	NA	Y	Y	NA	Y	Moderate
Charles et al. [32]	Y	N	N	P	N	N	N	P	Y	Y	N	NA	NA	NA	N	N	NA	Y	Low
Cummings et al. [33]	Y	N	N	Y	N	Y	Y	P	N	NA	N	NA	NA	NA	Y	N	NA	N	Low
Espejo-Antúnez et al. [34]	Y	P	Y	P	Y	Y	N	P	Y	NA	N	NA	NA	NA	Y	N	NA	Y	Moderate
Gattie et al. [35]	Y	N	N	P	Y	N	N	P	Y	NA	N	Y	NA	Y	Y	Y	Y	Y	Moderate
Kim et al. [36]	N	N	Y	P	N	Y	N	Y	Y	NA	N	NA	NA	NA	Y	N	NA	Y	Moderate
Mansfield et al. [28]	Y	Y	N	P	Y	N	N	P	Y	NA	N	N	NA	Y	Y	N	Y	Y	Low
Rodríguez-Mansilla et al. [37]	N	N	N	P	Y	Y	N	P	Y	NA	N	N	NA	N	N	N	Y	N	Critically low
Sánchez-Infante et al. [38]	Y	Y	N	P	Y	Y	N	P	Y	NA	N	Y	NA	Y	Y	Y	Y	Y	Moderate
Sousa Filho et al. [39]	Y	Y	N	Y	Y	Y	N	Y	Y	NA	Y	NA	NA	NA	Y	Y	NA	Y	Moderate
Tough et al. [40]	Y	N	N	P	Y	Y	Y	Y	P	NA	N	N	NA	Y	Y	N	N	N	Low
UPPER QUARTER																			
Al-Moraissi et al. [41]	Y	Y	N	P	P	N	N	P	Y	NA	N	Y	NA	Y	N	N	Y	Y	Low
Blanco-Diaz et al. [42]	Y	Y	N	P	Y	N	N	P	Y	NA	N	NA	NA	NA	Y	N	NA	Y	Moderate
Cagnie et al. [43]	Y	P	N	P	Y	Y	N	P	Y	NA	N	NA	NA	NA	Y	N	NA	Y	Moderate
Fernández-De-Las-Peñas. [44]	Y	Y	Y	PY	Y	Y	N	Y	Y	NA	Y	Y	NA	Y	Y	Y	Y	Y	High
Hall et al. [29]	Y	Y	N	P	Y	Y	N	Y	Y	Y	N	N	NA	Y	N	Y	N	Y	Critically low
Kietrys et al. [45]	N	P	N	P	N	Y	N	Y	Y	NA	N	Y	NA	Y	Y	Y	Y	Y	Moderate
Lew et al. [46]	N	Y	N	P	Y	N	Y	P	Y	NA	N	N	NA	Y	Y	N	N	Y	Low
Liu et al. [47]	Y	Y	N	P	Y	Y	N	P	Y	NA	N	Y	NA	Y	N	Y	Y	Y	Low
Machado et al. [48]	Y	Y	N	P	Y	Y	N	P	Y	NA	N	NA	NA	NA	Y	N	NA	Y	Moderate
Navarro-Santana et al. [49]	Y	Y	Y	P	Y	Y	Y	P	Y	NA	N	Y	NA	Y	Y	N	Y	Y	Moderate
Navarro-Santana et al. [30]	Y	Y	Y	P	Y	Y	Y	P	Y	NA	N	Y	NA	Y	Y	N	Y	Y	Moderate
Navarro-Santana et al. [50]	Y	Y	Y	P	Y	Y	Y	P	Y	NA	N	Y	NA	Y	Y	N	Y	Y	Moderate
Navarro-Santana et al. [51]	Y	Y	Y	P	Y	Y	Y	P	Y	NA	N	Y	NA	Y	Y	N	Y	Y	Moderate
Ong et al. [52]	Y	N	N	P	Y	Y	N	P	Y	NA	N	N	NA	N	Y	N	N	Y	Low
Pourahmadi et al. [53]	Y	Y	Y	Y	Y	Y	Y	Y	Y	NA	N	Y	NA	Y	Y	Y	Y	Y	High
Rodríguez-Huguet et al. [54]	Y	N	Y	N	Y	N	N	P	P	NA	N	N	NA	NA	Y	N	NA	Y	Critically low
Vier et al. [55]	Y	Y	N	P	Y	Y	Y	P	Y	NA	N	N	NA	Y	Y	N	N	Y	Low
LOWER QUARTER																			
He et al. [56]	Y	Y	N	P	N	N	Y	P	Y	NA	N	Y	NA	Y	Y	Y	Y	Y	Moderate
Hu et al. [57]	Y	N	Y	P	Y	Y	N	Y	Y	NA	N	N	NA	Y	Y	N	N	Y	Low
Khan et al. [58]	Y	P	N	P	Y	Y	Y	P	Y	Y	N	NA	NA	NA	N	N	NA	Y	Low
Liu et al. [59]	Y	Y	N	P	Y	Y	N	P	Y	NA	N	Y	NA	Y	Y	Y	Y	Y	Moderate
Llurda-Almuzara et al. [60]	Y	Y	N	P	Y	Y	N	P	Y	NA	N	Y	NA	Y	Y	Y	Y	Y	Moderate
Morihisa et al. [61]	N	N	N	P	Y	N	N	P	Y	NA	N	NA	NA	NA	Y	N	NA	Y	Moderate
Rahou-El-Bachiri et al. [62]	Y	Y	N	P	Y	Y	Y	P	Y	NA	N	N	NA	Y	Y	Y	Y	Y	Moderate
Ughreja et al. [63]	Y	P	N	P	Y	Y	Y	Y	Y	NA	N	N	NA	Y	N	N	N	Y	Critically low

Items 4, 9, 11 and 13 are critical items. Articles were scored high when they had no or a single non-critical weakness, moderate when they had more than one non-critical weakness, low when they had a single critical flaw with or without non-critical weaknesses, and critically low when they had more than one critical flaw with or without non-critical weaknesses. Abbreviations: N = No; Y = Yes; PY = Partial Yes; NA = Not Applicable.

**Table 2:** Recommendations for whole body and upper quarter regions.

Region	Recommendation
Whole body	A moderate recommendation can be made in favor of DN for patients with MSK pain to decrease pain intensity in all body regions. Results from all included reviews and meta-analyses are in line at short-term: there is superiority of DN interventions over sham/placebo or no intervention for reducing pain and improving functional outcomes in MSK pain. DN is at least equally effective compared to other interventions (e.g., MT or other needling interventions) for reducing pain. However, there is insufficient evidence to evaluate the effect of DN over other interventions for improving functional outcomes. The use of DN as a component to PT interventions is supported. The evidence on long-term effects is still limited and should be interpreted with caution.
TMJ	A weak recommendation can be made for the use of DN for pain reduction and increased range of motion in TMJ in the short-term. Studies suggest this technique to be a cost-effective alternative for WN, although evidence is limited and of low methodological quality. Further research is necessary.
Neck	For neck pain, a strong recommendation can be made for the superiority of DN for the reduction of pain intensity at short-term, compared to sham/placebo. DN can be equally effective as other treatments, except for WN. Combined interventions (DN + other interventions) can be recommended for the improvement of pain and disability in the short-term. There is a moderate recommendation that DN reduces disability and improves strength and functionality versus control interventions in the short-term. DN cannot be recommended for improvements in range of motion, as the evidence is limited and conflicting.
Shoulder	A moderate recommendation based on three SRs can be made. DN can be an equally effective technique in the short-term for reduction of pain and disability compared to sham/control interventions/MT. At mid-term follow-up, WN or other treatments should be preferred. No conclusions can be drawn for range of motion or strength. All reviews recommend the use of DN for treating MSK pain. DN is safe and effective in reducing pain and disability in subacromial syndrome and non-traumatic shoulder pain.

## Data Availability

Not applicable.

## Supplementary Materials

The following supporting information can be downloaded at: https://www.mdpi.com/article/10.3390/jcm12031205/s1, Tables S1 and S2: PICOS table and search strategy; Table S3: Reference list of primary studies; Tables S4–S6: Overlap for pairs of reviews; Table S7: Citation Matrix.

## Appendix A

**Table A1:** Evidence table.

Author (Year) Country Study Type	Number of RCTs (Year) Total Sample	Body Region	Intervention	Comparison (COM)	Outcome Measures Follow-Up	Results (Number of RCTs)	Adverse Events (AE)	Remarks
WHOLE BODY								
Boyles et al., (2015) [31] USA SR	19 RCTs (2002–2014) N total = 1031	Neck (9) TMJ (2) Lower extremity (3) Shoulder (1) Elbow (1) Lower back (1) Gluteal region (1) NR (1)	DN (17) Superficial DN + stretching (2) Symptomatic deep DN (1)	Sham (14) AC (2) Stretching (2) No intervention (2) WN (2) PENS (1) Medication (1) MSN (1) MT (1)	Pain intensity: VAS, SFMPQ, NPRS, NPQ Strength: NR ROM: custom device, SLR, goniometry, MMO Disability/Functionality: NDI, FFI, WOMAC, DASH-21 Follow-up: NR (Range: immediate—6 m)	DN > stretching or PENS DN ≥ MT 2. No influence of DN 3. Conflicting results 4. Conflicting results	NR	Heterogeneity among patient characteristics and protocols limits comparison between studies.
Charles et al., (2019) [32] USA SR	23 RCTs (1993–2015) N total = 1217	Neck (13) Shoulder (6) Knee (1) Ankle (1) NR (4)	DN (17) DN + spray + stretching (1) DN + stretching (2) DN with paraspinal needling (1) DN + PT (2)	Control Intervention (7) Sham (8) MT (5) WN (2) + stretching (2) PT (3) AC (2) Stretching (1) Laser + stretching (1)	Pain intensity: NPRS, VAS, MPQ, NPQ ROM: RMI Follow-up: NR (Range: immediate—12 w)	DN > COM (11) DN = COM (5) DN < COM (4) 2. DN > COM (4) DN = COM (2) DN < COM (1)	NR	A lack of standardized guidelines in the location of trigger points affected the reliability of the physical examination and subsequent treatment results.
Cummings et al., (2001) [33] UK SR	5 RCTs (1989–1997) N total = 532	Neck (4) Lower back (1)	DN (4) DN + WN (1)	Sham (3) WN (2) WN + sham (1)	Pain intensity: VAS Follow-up: NR (Range: 24 h–17 w)	WN is not superior to DN (3)	NR	Small sample size. One study was not correctly randomized and suffered major loss of follow-up.
Espejo-Antúnez et al., (2017) [34] Spain, Portugal SR	15 RCTs (2002–2015) N total = 761	Neck (12) TMJ (1) Shoulder (1) Knee (1)	DN (15)	Sham (8) WN (5) MT (2) No intervention (1) Pharmaco-logical intervention (1) Laser (1) AC (1) DN with paraspinal needling (1)	Pain intensity: VAS ROM: NR Disability: WOMAC, NPQ QoL: NR Pain Pressure Threshold Follow-up: NR (Range: immediate-6m)	DN > SHAM/No intervention/WN DN ≥ MT DN = Pharmacological intervention 2. DN > Sham/placebo DN = WN/pharmacological intervention/MT 3. DN > Sham/Placebo DN = MT 4. DN = WN/pharmacological intervention 5. Conflicting evidence	NR	Heterogeneous DN protocols, only 5 studies specified the use of deep dry needling.
Gattie et al., (2017) [35] USA, Australia SR and MA	13 RCTs (2003–2015) N total = 723	Neck (6) Shoulder (1) Lower back (1) Knee (1) Ankle (1) NR (3)	DN (10) DN + PT (3)	MT (4) Control (4) PT (3) Sham (2) + PT (1) Stretching (1) PENS (1)	Pain intensity: VAS, MPQ, NPRS Disability/Functionality: NDI, SF-36, WHOQOL-BREF, NPQ, FAAM, DASH, ADLs Follow-up: - short-term (immediate—12 w) - long-term (6–12 m)	Short-term: DN > control, sham (SMD = −0.7; 95% CI: −1.06, −0.34) DN > other treatments (SMD = −0.43; 95% CI: −0.77, −0.10) Long-term: DN > control, sham (SMD = −0.26; 95% CI: −0.58, 0.06) 2. Short-term: DN > control, sham (SMD = −0.44; 95% CI: −0.85, −0.04) DN = other treatments (SMD = −0.01; 95% CI: −0.49, 0.47) Long-term: DN > control, sham (SMD = −0.32; 95% CI: −0.62,−0.02)	NR	Very low to moderate quality evidence. High heterogeneity in 5 out of 8 meta-analyses. Significant heterogeneity among studies (i.e., sample, comparison and follow-up).
Kim et al., (2012) [36] South Korea SR	4 RCTs (1980–2003) N total = 166	Neck (2) Shoulder (1) Lower back (1)	DN (2) + IMES (2)	Medication (1) PT (1) Sham (1) DN (1) IMES (1)	Pain intensity: VAS, MPQ ROM: NMLS Follow-up: - short-term (immediate—4 w)	Short-term: IMS > Sham (1) IMES = medication (1) IMES > DN (1) IMS + PT > PT (1) 2. DN > sham (1) IMES = medication (1) IMES > DN (1)	There were no adverse effects related to IMS or sham.	Inconclusive evidence in support of DN and IMES. Too many important caveats (small sample size, only one RCT for each condition) exist to draw firm conclusions.
Mansfield et al., (2019) [28] USA SR and MA	21 RCTs (2001–2018) N total = 977	Neck (2) Upper extremity (8) Shoulder (4) Knee (4) Ankle (4)	DN (7) DN + stretching (2) DN + PT (1) AC (11)	Stretching (2) PT (2) Placebo (2) Sham (6) DN (3) MT (1) No treatment (6) Laser (with/without water massage) (1) US (1) Electro AC (1)	Strength: CMS, grip strength, isometric cervical force production Follow-up: - short-term (immediate—3 m)	Short-term: Cervical: DN > COM (moderate strength of evidence in non-specific neck pain) No significant differences in other body regions. No information on long-term effects.	NR	/
Rodríguez- Mansilla et al., (2016) [37] Spain SR and MA	19 RCTs (2002–2012) N total = 852	Neck (9) Shoulder (4) Gluteal region (2) TMJ (2) Elbow (1) NR (1)	DN (14) DN + stretching (3) DN + PT (2)	Sham (6) WN (4) PT (1) No intervention (1) Stretching (1) + US (1) MT (1)	Pain intensity: VAS, SFMPQ ROM: RMI Follow-up: - immediate - short-term (3–4 w)	Immediate: DN > placebo (95% CI: −3.21, 0.42) DN > control (95% CI: −14.70, −3.56) DN < other treatment (95% CI:−0.040, 5.48) Short-term: DN < other treatments (95% CI: 0.78, 7.68) 2. Immediate: DN > placebo (95% CI: 1.60, 2.41) DN < other treatments (95% CI:−1.84, −0.99) Short-term: NR	NR	9 out of 19 included studies were not included in MA
Sánchez-Infante et al., (2021) [38] Spain SR and MA	42 RCTs (2007–2020) N total = 3967	Neck (16) Shoulder (5) Elbow (2) Abdomen (1) Lower back (3) Hip (1) Gluteal region (1) Knee (5) Ankle (1) Heel (3)	DN (29) DN + PT (8) DN + stretching (3) DN + ESWT (1) DN + MT + PT (1)	Sham (11) + PT (1) PT (9) + stretching (1) + MT (1) + DN (1) MT (8) ESWT (4) Kinesiotaping (2) Stretching (2) Control (1) Medication (1) Peppering (1) DN + US (1) No intervention (1) TENS (1) PENS (1)	Pain intensity: VAS, NPRS Follow-up: - immediate (0–72 h) - short-term (1–3 w) - mid-term (4–12 w) - long-term (13–24 w)	Immediate: DN (alone or with other therapy) > placebo/other therapy Low quality evidence for a large effect (SMD = −0.81; 95% CI = −1.21 to −0.40; n = 1542; *p* < 0.000) Short-term: DN (alone or with other therapy) > placebo/other therapy Moderate quality evidence for a moderate effect (SMD = −0.69; 95% CI= −1.02 to −0.35; n = 808; *p* < 0.000) Mid-term: DN (alone or with other therapy) > placebo/other therapy Low quality evidence for a large effect (SMD = −0.85; 95% CI= −1.30 to −0.40; n = 1261; *p* < 0.000) Long-term: DN (alone or with other therapy) > placebo/other therapy Low quality evidence for a large effect (SMD = −0.81; 95% CI= −1.64 to −0.03; n = 365; *p* = 0.06)	NR	An important limitation is the high heterogeneity of the analyzed studies.
Sousa Filho et al., (2021) [39] Brazil SR	6 RCTs (2008–2021) N total = 384	Elbow (2) Heel (2) Headache (1) Gluteal region (1)	DN (6)	Corticosteroid Injection (6)	Pain: VAS, NPRS Disability & function: PSFS, DASH, PRTEE, FFI Follow-up: - short-term (≤6 weeks) - mid-term (7–23 weeks) - long-term (≥24 weeks)	1 & 2: short- and mid-term: CSI > DN for plantar fasciitis & lateral elbow pain DN = CSI for myofascial pain and gluteal tendinopathy Long-term: DN > CSI for plantar fasciitis & lateral elbow pain DN = CSI for myofascial pain and gluteal tendinopathy	Reported for primary studies	Very low quality evidence (GRADE)–insufficient evidence.
Tough et al., (2009) [40] UK SR and MA	7 RCTs (1997–2007) N total = 564	Neck (3) Low back (2) Shoulder (1) Gluteal region (1)	DN (4) DN + PT (1) DN + home exercise (1) EMG needling (1)	Sham (6) PT (1)	Pain intensity: VAS Follow-up: - short-term (Range: immediate—3 w)	Short-term: DN not significantly better than SHAM DN > usual care	NR	4 out of 7 studies included in meta-analysis. Marked heterogeneity was observed.
UPPER QUARTER								
Al-Moraissi et al., (2019) [41] Yemen SR and MA	21 RCTs (1997–2019) N total = 515	TMJ (21)	DN (7) AC (4) WN (10)	WN (12) Placebo (9) Laser (1) No treatment (1)	Pain intensity: VAS PPTs ROM: MMO Follow-up: (Range: Immediate—6 months post-treatment)	Short-term: DN: no sign. differences compared to placebo or WN at short-term (Results favour DN over placebo, but not over PRP and local anaesthesia) Mid-term: No sign. Diff between DN, WN or placebo 2. No differences of DN compared to WN or placebo 3. Sign. Improvement of MMO of WN (Lidocaine) and DN vs. placebo.	NR	/
Blanco-Diaz et al., (2022) [42] Spain SR	9 RCTs (2016–2021) N total = 421	Shoulder (9): Subacromial Syndrome	DN (4) DN + PT (2) DN + MT (2) PT (1)	DN + PT (1) PT (2) MT (2) Post-isometric relaxation (1) DN + post-isometric relaxation (1) DN (1)	Pain intensity: VAS, NPRS ROM: N Disability/Functionality: DASH, PSS, GROC, SPADI QoL: EuroQol-5D, QUALY PPTs - short-term (3 m) - long-term (6 m)	DN: sign. Improvement. No relevant differences at follow-up compared to COM 2. DN < COM (1) DN > COM (3) 3. DN = COM (5) DN > COM (1) 4. DN > COM (1) 5. DN = COM (4)	Reported for the individual primary studies	Study characteristics of one study were not reported. Heterogeneous DN protocols (e.g., number and length of sessions).
Cagnie et al., (2015) [43] Belgium SR	15 RCTs (1994–2013) N total = 814	Neck (15)	IC (7) DN (8)	Sham (8) WN (3) MT (2) Stretching (2) US (1) PT (1) Laser (1) INIT (1) MET (1) No intervention (1) DN with paraspinal needling (1) MSN (1)	Pain intensity: VAS ROM: NR Disability/Functionality: NDI QoL: NHP PPT NR (Range: immediate—12 w)	Decreases after DN DN > COM (1) DN < WN (1) DN < MSN (1) 2. DN = CI (6) 3. DN > CI (1) 4. DN = WN/Medication (1) 5. Sign increase (3) of PPT; DN > COM (3) DN = COM/WN (3)	NR	Heterogeneity in the results. Heterogeneous DN protocols (i.e., sessions and frequency, different muscles needled). Results should be interpreted with caution.
Fernández De Las Peñas (2021) [44] Spain SR and MA	8 RCTs (2010–2020) N total = 631	Neck (8)	DN + other interventions (OI): standardized PT (1) exercise therapy (1) passive stretching (2) MT (1) guideline based PT (1) PENS (1) Pain neuroscience education (1)	Sham DN + standardized PT (1) Exercise therapy (1) Manual therapy (1) Passive stretching (2) Guideline based PT (1) DN alone (2) Usual Care (1)	Pain intensity: NPRS, VAS Disability: NDI PPT Range Of Motion Pain Catastrophizing: PCS - short-term (0–12 weeks) - mid-term (12–24 weeks) - long-term (24+ weeks)	DN + OI > COM short and mid-term 2. DN + OI > COM short-term DN + OI = COM at mid- and long-term 3. DN + OI: increases PPTs, compared to OI alone, short-term 4. DN + OI = COM 5. DN + OI > COM mid- & long-term, not short-term	7/8 trials reported information about minor AE: post-needling soreness No serious adverse effects were reported.	Although the methodological quality of the included trials was high, the inconsistency (heterogeneity) and imprecision of the results downgraded the overall levels of evidence.
Hall et al., (2018) [29] New Zealand SR and MA	10 RCTs (2004–2016) N total = 496	Shoulder (11)	DN (10) Active + latent DN (1)	Sham (3) PT (3) + DN (1) No intervention (1) Electro-AC (1) DN + electro-AC (1) MT (1) Only active DN (1)	Pain intensity: NPRS, VAS Disability/Functionality: CMS, DASH ROM Strength: subsection of CMS, grip strength PPT Follow-up: NR (Range: immediate—12 w)	DN > COM (7) DN = COM (2) 2. DN > Sham (1) DN + PT > PT (1) DN = MT (1) 3. DN > No treatment (1) DN + PT > PT (2) DN vs. electro-AC: conflicting results (1) 4. DN + PT > PT (2) No diff. between DN of active + latent points vs. DN of active MTrPs alone. 5. DN > no treatment/PT for local and distant sites (FU-1 week)	2/11 trials reported AE: Bruising, bleeding & pain. No serious adverse effects were reported.	Significant heterogeneity among studies (e.g., different muscles needled, frequency and number of sessions, control group interventions, outcome measures and follow-up), making pooling of data difficult.
Kietrys et al., (2013) [45] USA SR and MA	12 RCTs (1994–2010) N total = 696	Neck (7) Shoulder (3) Upper quarter (2)	DN (9) DN + stretching (3)	Sham (5) WN (3) Stretching (2) Acupuncture (2) (Sham) Laser (2) DN to random points/ contralateral side (3) Rehabilitation (2) IMS (1)	Pain intensity: VAS Follow-up: - immediate - short-term (4 w)	Immediate: DN > sham or control (SMD = 1.06; 95% CI: 0.05, 2.06) DN < WN/acupuncture (SMD = −0.64; 95% CI: −1.21, −0.06) Short-term: DN > sham or control (SMD = 1.07; 95% CI: −0.21, 2.35) DN < WN/acupuncture/laser (SMD = −0.07; 95% CI: −1.39, 1.26)	NR	High heterogeneity in the results of the MA
Lew et al., (2021) [46] USA SR and MA	6 RCTs (2014–2017) N total = 241	Neck (6)	DN (4) DN + stretching (2)	MT (6)	Pain intensity: VAS, NPRS Disability: NDI Follow-up: - short-term (7–28 d)	Short-term: DN = MT (SMD = 0.41; 95% CI: −0.18, 0.99) 2. Short-term: DN = MT (SMD = −0.66; 95% CI: −1.33, 0.02)	Not reported in the primary studies	
Liu et al., (2015) [47] China SR and MA	20 RCTs (1994–2014) N total = 839	Neck (12) Neck + shoulder (8)	DN (13)	Sham (6) WN (3) IMES (2) PT (1) MT (1)	Pain intensity: VAS, NPRS Follow-up: - short-term (immediate—3 d) - mid-term (9–28 d) - long-term (2–6 m)	Short-term: DN > sham DN = WN DN = other treatments Mid-term: DN > sham DN < WN DN < other treatments Long-term: DN = sham DN = WN DN = other treatments	NR	A lack of a substantial number of studies comparing DN with control/sham in the short term.
Machado et al., (2018) [48] Brazil SR	18 RCTs (2002–2016) N total = 368 (3 studies did not report n)	TMJ (16) TMJ + neck (2)	DN (7)	WN (4) Sham (2) Medication (1)	Pain intensity: VAS, SSI ROM: MMO PPT Follow-up: NR (Range: immediate—6 m)	DN = WN (6) DN > medication (1) 2. DN > sham (1) DN = Medication (1) 3. DN > Sham	AE were only reported in studies for WN: pain, paralysis, difficulty in swallowing, discomfort in chewing.	
Navarro-Santana et al., (2020) [49] Spain SR and MA	28 RCTs (2004–2020) N total = 1319	Neck (24) Neck and shoulder (4)	DN (24) DN + stretching (3) DN + home exercise (1)	MT (10) Sham (6) Kinesiotaping (4) ESWT (2) Usual care (2) DN + neuroscience education (1) No intervention (1) Soft tissue techniques (1) PENS (1)	Pain intensity: NR Disability: NR ROM: NR PPT Follow-up: - immediate - short-term (12–24 w) - mid-term (>24 w)	Immediate: DN > sham/placebo/no intervention/other needling Short-term: DN > COM; DN = Other interventions Mid-term: no sign. diff. 2. Immediate: NR Short-term: DN > COM; DN = MT/other PT interventions Mid-term: no sign. diff. 3. Immediate: not significant; Short-term: not significant; Mid-term: NR 4. No sign. overall effect immediately and at short-term. Immediate subgroup effect: DN > sham/placebo/no intervention/other needling Mid-term: NR	50% of trials reported post-needling soreness as main minor AE. There were no serious AE.	Heterogeneous DN and COM protocols.
Navarro-Santana et al., (2020) [30] Spain SR and MA	7 RCTs (2011–2019) N total = 320	Elbow (7)	DN (3) DN + PT (2) Tendon-DN (1) DN + ESWT + home exercise + cold application (1)	PT (3) Sham (1) NSAID + bracing (1) MT (1) ESWT + home exercise + cold application (1)	Pain intensity: VAS, pain PRTEE Disability: PRTEE Grip strength: NR PPT Follow-up: - immediate (0–72 h) - short-term (0–12 w) - long-term (>12 w)	Immediate: not significant Short-term: DN > COM Long-term: DN > COM 2. Immediate: NR Short-term: DN > COM Long-term: DN > COM 3. Immediate: not significant Short-term: DN > COM Long-term: NR 4. Immediate: NR Short-term: DN > COM Long-term: NR	6/7 trials did not report AE. One trial reported a minor event: local hemorrhage.	High heterogeneity between the trails should be taken into account when interpreting the results.
Navarro-Santana et al., (2021) [50] Spain SR and MA	6 RCTs (2014–2019) N total = 381	Shoulder (6)	DN (3) DN + exercise (1) DN + US (1) DN + personalized treatment (1)	SHAM (1) Exercise (1) Personalized treatment (1)	Pain intensity: VAS, NPRS Disability: Constant Murley Score, SPADI, DASH Follow-up: - short-term (0–1 month) - mid-term (1–3 months) - long-term (3–6 months)	Short-term: DN > COM (small effect) Mid-term: no sign. differences 2. Short-term: DN > COM (large effect) Mid-term: no sign. differences	Post-needling soreness (25% of patients)	Serious heterogeneity between trials. Long-term effects were only based on 1 trial.
Navarro-Santana et al., (2022) [51] Spain SR and MA	7 RCTs (1994–2019) N total = 426	Neck (7)	DN (4) DN + home exercise (2) DN + stretching (1)	WN (7) WN + home exercise (2) WN + stretching (1)	Pain intensity: VAS, NPRS ROM: NR Disability: NDI PPT Follow-up: - immediate - short-term (1–12 w) - mid-term (12–24 w)	Immediate: not significant (SMD = − 0.58; 95% CI: −1.20, 0.04) Short-term: DN < WN (*p* < 0.001) Mid-term: not significant (SMD = − 0.28; 95% CI: −0.64, 0.08) 2. No significant differences 3. No significant differences (SMD = 0.90; 95% CI: −3.09, 4.89) 4. Immediate: No sign. differences	Minor AE for WN: post-needling soreness, muscle pain, and discomfort, paresthesia, fatigue, headache, hemorrhage, transient flare reaction, and dizziness Minor AE for DN: post-needling soreness, pain, dis-comfort, a transient flare reaction.	6/7 studies included in meta-analysis.
Ong et al., (2013) [52] New Zealand, UK SR and MA	5 RCTs (1994–2010) N total = 266	Neck (5)	DN (2) DN + home exercise (1) DN + stretching (1) DN + laser therapy (1)	WN (2) WN + home exercise (1) WN + stretching (1) Sham (1)	Pain intensity: VAS QoL: NHP Follow-up: - immediate - short-term (1–4 w) - long-term (3–6 m)	Immediate: not significant Short-term: DN = WN (favoring WN) Long-term: DN = WN (favoring DN) 2. No significant differences (based on 1 study)	NR	The risk of bias on all RCT’s were generally unclear.
Pourahmadi et al., (2021) [53] Iran SR and MA	11 RCTs (1994–2019) N total = 685	Headache (11)	DN (11)	Sham (4) Sham + Medication (1) C1-C2 SNAGs (2) MT (2) WN (4) No intervention (1)	Pain intensity: VAS, NPRS ROM: NR Disability: HDI QoL: SF-36 Headache frequency Follow-up: - short-term (<3 m)	Short-term: DN = COM (TTH: SMD = −1.27; 95% CI: −3.56, 1.03; CGH: SMD = −0.41; 95% CI:−4.69, 3.87; Mixed headache: SMD = 0.03; 95% CI: −0.42, 0.48) 2. Short-term: DN not significantly better than sham (TTH: SMD ≥ −0.48; 95% CI:−2.44, 1.48) DN > SNAGs (CGH: SMD ≥ 0.65; 95% CI: 0.19, −1.52) 3. Short-term: DN > COM (TTH: SMD = −2.28; 95% CI: −2.66, −1.91; CGH: SMD = −0.72; 95% CI: −1.09, −0.34) 4. DN > COM (TTH: SMD = −2.45; 95% CI: −2.85, −2.05) 5. DN > Sham for decreased frequency (TTH: SMD −1,79; 95% CI: −2.14, −1.41; CGH: SMD = −0.94; 95% CI: −1.77, −0.12)	Minor AE: pain, fear, gastrointestinal discomfort, euphoria.	These results should be interpreted with caution due to a lack of high-quality studies. Heterogeneous DN techniques.
Rodriguez-Huguet et al., (2021) [54] Spain SR	11 RCTs (2002–2021) N total = 807	Neck (11)	DN (4) DN + stretching (2) DN + MT (1) DN + MT + exercise (2) DN + PENS (low vs. high freq) (1) DN + stretching + education (1)	Needle acupuncture/sham laser acupuncture (1) TrP MT (2) Passive stretching (1) Shock wave (1) SHAM + MT (1) MT (mobilization) + exercise (1) SHAM + MT + exercise (1) DN + PENS (low vs. high freq) (1) TENS + Microwave + stretching (1)	Pain: VAS, NPRS PPT ROM Strength Disability: NDI, NPQ Perceived effects Follow-up: NR	- Positive outcomes were achieved in the short- term and in the follow-up performed between three and six months. - Effects seemed to be limited in very long-term follow-ups, such as one year.	NR	The variability among studies could make it difficult to determine conclusions.
Vier et al., (2019) [55] Brazil SR and MA	7 RCTs (1997–2015) N total = 199	TMJ (7)	DN (4) DN + pain education (1) DN + LI + stretching (1) DN + sham PI (1)	Sham (3) Sham + WN (1) Sham + pain education (1) WN + DN (1) Medication (1) Laser + stretching (1)	Pain intensity: VAS, NPRS ROM: MMO PPT Follow-up: - short-term (<3 m) - mid-term (3–6 m - long-term (>6 m)	Short-term: DN = Sham (SMD = 0.30; 95% CI:−0.83, 1.43) DN > other COM (SMD = −0.74; 95% CI:−1.25, −0.22) Mid and long-term: NR 2. Short-term: DN = Sham (SMD = 0.12; 95% CI:−3.04,2.80) Mid and long-term: NR 3. Short-term: DN > Sham (SMD = 0.56; 95% CI: 0.31, 0.81)	NR	Study quality was overall very low. 5 out of 7 studies included in meta-analysis.
LOWER QUARTER								
Khan et al., (2021) [58] Pakistan SR	10 RCTs (2005–2020) N total = 466 (one study did not report n)	General: Knee (3) Heel (4) Lower extremity (3)	DN (7) DN + PT (2) DN + MT (1)	Sham (5)+ PT (1) PT (2) MT (1) PENS (1)	Pain intensity: VAS, NPRS, FHSQ (pain subscale) ROM: NR QoL: EuroQol-5D-5L Follow-up: - short-term (immediate—12 w)	Short-term: DN > COM (6) DN = COM (3) 2. DN = COM (4) 3. NR	NR	No reported positive effects of DN on depression, anxiety and muscular strength.
Morihisa et al., (2016) [61] USA SR	6 RCTs (1983–2014) N total = 301	General: Lower back (2) Upper body (1) Gluteal region (1) Knee (1) Heel (1)	DN (5) DN + stretching (1)	Sham (6) No intervention (1) Stretching (1)	Pain intensity: VAS, SFMPQ ROM: NR QoL: SF-36 Disability: WOMAC, FHSQ Follow-up: - short-term (immediate—3 m) - long-term (3–6 m)	Short-term: DN > COM Long-term: not significant 2. No significant diff. 3. NR 4. No significant diff.	NR	No MA conducted. Heterogeneous outcome measures.
Hu et al., (2018) [57] China SR and MA	16 RCTs (1989–2016) N total = 1274	Lower back (16)	DN (16)	AC (9) + DN (2) WN (3) SHAM (2) PT (1) DN + education (1) Laser (1)	Pain intensity: VAS Disability: ODI, RDQ QoL: TSK Follow-up: - immediate - short-term (<3 m)	Immediate: DN > SHAM/AC/WN/PT DN = WN/DN + education DN < laser/DN + AC Short-term: DN > SHAM (SMD = −1.05; 95% CI:−1.70, −0.40) DN = AC (SMD = −0.47; 95% CI: −1.04, 0.09) DN > PT DN < laser 2. Immediate: DN > SHAM (SMD = −1.70; 95% CI:−2.59, −0.81) DN > AC (SMD = −0.63; 95% CI:−0.99, −0.26) DN = WN/DN + education DN < laser Short-term: DN = SHAM (SMD = −0.58; 95% CI:−1.19, 0.04) DN = AC (SMD = −0.10; 95% CI: −0.65, 0.45) DN > PT DN < laser 3. NR	3/16 trials reported AE: sticking of the needle, deterioration of symptoms, increasing pain and complaints of fever and chills.	Conflicting results led to uncertainty whether DN was superior to these other treatments (i.e., laser, WN, PT).
Liu et al., (2018) [59] China SR and MA	11 RCTs (2004–2016) N total = 682	Lower back (11)	DN (11)	AC (5) Sham (3) WN (1) Laser (1) PT (1) DN + education (1)	Pain intensity: VAS Disability: ODI, RDQ Follow-up: - immediate - short-term (<3 m)	Immediate: DN > COM Short-term: No significant differences 2. Immediate: DN > COM Short-term: No significant differences	NR	
Rahou-El-Bachiri et al., (2020) [62] Spain SR and MA	10 RCTs (2008–2020) N total = 473	Knee (10)	DN (7) DN + MT + exercise (1) DN + exercise (1) Superficial DN (1)	Sham (2) Sham + PT (3) PT (3) Ultrasound (1) AC (1) MT + PT (1)	Pain intensity: VAS, NPRS Disability: WOMAC, KOOS Follow-up: - short-term (0–10 w) - mid-term (10–20 w) - long-term (>20 w)	Short-term: DN >COM Mid-term: not sign. Long-term: not sign. 2. Short-term: DN > COM Mid-term: not sign. Long-term: not sign.	Minor AE: post-needling soreness and hemorrhages. No serious adverse events reported.	Overall low quality of evidence. Heterogeneous DN protocols (i.e., sessions and frequency, different muscles).
Ughreja et al., (2021) [63] India SR and MA	9 RCTs (2007–2019) N total = 778	Knee (9)	PST (2) + home exercise (1) + MT + PT (1) DN + PT (2) DN (1) IMES (2)	Sham (5) Sham + PT (2) MT + PT (1) TENS + home exercise (1)	Pain intensity: VAS, WOMAC Disability/Functionality: WOMAC Follow-up: - Immediate - long-term (3–9 m)	Immediate: PST > COM IMES > COM Long-term: PST > COM Immediate: PST > COM Long-term: PST > COM	1/9 trials reported minor AE: muscle soreness, bruising, headache, sweating.	Significant heterogeneity among studies (i.e., number of sessions, control group interventions, out- come measures and follow-up) making pooling of data difficult.
He et al., (2017) [56] China SR and MA	7 RCTs (2011–2017) N total = 417	Heel (7)	DN (2)	Sham (1) NR (1)	Pain intensity: VAS Follow-up: - short-term (1 m) - mid-term (6 m) - long-term (12 m)	overall: DN > COM Short-term: DN > COM Mid-term: DN > COM Long-term: DN > COM	3/7 trials reported minor AE: needle site pain or subcutaneous bleeding.	Adverse events were similar between DN and CI. Marked heterogeneity was observed.
Llurda-Almuzara et al., (2021) [60] Spain SR and MA	6 RCTs (2014–2020) N total = 395	Heel (6)	DN (6)	WN (2) ESWT (2) Stretching + massage (1) Sham (1)	Pain intensity: VAS, FFI Disability/Functionality: FHSQ, FFI Follow-up: - short-term (<4 w) - mid-term (4–12 w) - long-term (>12 w)	Overall: not sign; Short-term: DN > COM Mid-term: NR Long-term: DN > COM 2. Overall: DN > COM Short-term: not sign. Mid-term: not sign. Long-term: DN > COM	Minor AE: post-needling soreness, subcutaneous bleeding, bruising, exacerbation of symptoms. No major AE were reported.	/

Abbreviations: AC: acupuncture; ADLs: activities of daily living; BDI: Beck depression inventory; CGH: cervicogenic headache; CMS: Constant-Murley score; Comparison: COM; DASH: disabilities of the arm, shoulder and hand questionnaire; EMG: electromyography; ESWT: extracorporeal shockwave therapy; EuroQol-5D-5L: EuroQol five-dimension-5-level scale; FAAM: foot and ankle ability measure; FFI: foot function index; FHSQ: foot health questionnaire; GROC: global rating of change functional outcome score; HDI: headache disability index; IC: ischemic compression; IMES: intramuscular electrical stimulation; INIT: integrated neuromuscular inhibition technique, combination of MET, IC, and strain-counterstrain; KOOS: knee injury and osteoarthritis outcome score; m: months; Medication: flurbiprofen, paracetamol, metoprolol; MET: muscle energy techniques; MMO: maximal mouth opening; MSN: mini scalpel needling; MT: manual therapy, ischemic compression, manipulation, soft tissue techniques; NDI: neck disability index; NHP: Nottingham health profile; NMLS: neck movement limitation score; No intervention: waiting list, no treatment, wait and see; NPQ; neck pain questionnaire; NPRS: numeric pain rating scale; NR: not reported; NSAID: non-steroidal anti-inflammatory drug; OI: Other Interventions; ODI: Oswestry disability index; PENS: percutaneous electrical nerve stimulation; PSFS: Patient-Specific Functional Scale; PRTEE: patient rated tennis elbow evaluation; PSS: Penn shoulder score; PST: periosteal stimulation; PT: physical therapy, mobilisation, exercise, massage; QoL: quality of life; QUALY: quality-adjusted life-year; RDQ: Roland Morris disability questionnaire; RMI: Rivermead mobility index; SF-36: 36-item short form survey; (SF)MPQ: (short form) McGill pain questionnaire; Sham: superficial needling, not in TrP, sham laser, sham ultrasound, placebo; SNAGs: sustained natural apophyseal glides; SPADI: shoulder pain and disability index; Stretching: active, passive or self-stretching; DN: trigger point dry needling; TENS: transcutaneous electrical nerve stimulation; TMJ: temporomandibular joint; TSK: Tampa scale for kinesiophobia; TTH: tension- type headache, VAS: visual analogue scale; w: weeks; WHOQOL-BREF: short form of the world health organization quality of life questionnaire; WN: wet needling, procaine, lidocaine, corticosteroid, botulin toxin a, flurbiprofen injection; WOMAC: western Ontario and McMaster universities osteoarthritis index.
