# Supplementary material for: Clinical Effectiveness of Dry Needling in Patients with Musculoskeletal Pain—An Umbrella Review
Source: J Clin Med. 2023 Feb 2;12(3):1205. doi: 10.3390/jcm12031205 (PMC9917679; doi:10.3390/jcm12031205)
Supplement: Supplementary file 1 [file jcm-12-01205-s001.zip › Suppl. Material_Table S1-S2_Search Strategy.pdf]

Table S1. PICOS table.

P	• Patients with musculoskeletal disorders.<br>• Patients aged 18-65 Y<br>• Pain can be acute, subacute chronic or recurrent<br>• No metabolic/neurological/cardiovascular pathology or widespread pain syndromes i.e. fibromyalgia, chronic fatigue syndrome.<br>(Fibromyalgia, a chronic and complex pain syndrome, typically presents with symptoms of diffuse body pain frequently involving the spinal region. Whereas myofascial pain may involve only one or two regions of the body, the pain of fibromyalgia is widespread and accompanied by multiple tender points, which differ from trigger points histologically and lack the typical trigger point pain referral pattern.)
I	• At least one DN session (as a stand-alone intervention or combined with another treatment modality [e.g., therapeutic exercise])<br>• DN = intramuscular technique according to the MTrP approach, an acupuncture needle is inserted directly into an MTrP.<br>• Deep dry needling and superficial dry needling<br>• DN with or without paraspinal needling
C	Compared to:<br>1. Placebo/sham/no intervention<br>Placebo: According to the definition by Shapiro & Morris, 'a *placebo* is defined as any therapy or component of therapy used for its nonspecific, psychological, or psychophysiological effect, or that is used for its presumed specific effect, but is without specific activity for the condition being treated'.<br><br>In the case of a placebo for dry needling interventions, it seems necessary to contact the skin and therefore result in some physiological stimulation.<br>We therefore prefer to use the term sham rather than placebo.<br>Since the term placebo has been described, this will be added to the search.<br><br>a. Sham needling:<br>i. Nonpenetrating techniques (e.g. blunt needle, or commercial sham devices like the Streitberger and Kleinhenz sham or the Park sham)<br>ii. Penetrating sham groups:<br>1. Inserting the needles subcutaneously only (i.e., superficial dry needling above trigger points (TrP SDN)<br>2. Inserting the needle away from trigger points (Non-TrP SDN))<br>3. Inserting needles into muscle but away from trigger points (Non-TrP DN)<br>b. Control: No intervention/waiting list<br><br>2. Other interventions<br>a. Manual therapy (Mobilisation, manipulation, manual ischemic compression techniques)<br>b. Physical therapy (Exercise therapy, stretching…)<br>c. Massage/soft tissue techniques<br>d. Wet Needling<br>e. PENS<br>f. Medication/Pharmacological intervention<br>g. Acupuncture<br>h. Extracorporeal shockwave therapy (ESWT)<br>i. Laser therapy<br>j. Intramuscular (electrical) stimulation (IMES/IMS)<br>k. Ultrasound<br>l. Kinesiotaping

	m. Transcutaneous Electrical Nerve Stimulation (TENS)<br>n. Usual care<br>o. (Neuroscience) education<br>p. Heat/cold therapy
O	At least one clinical outcome in the domain of:<br>1. Pain<br>2. Physical functioning (e.g. active and passive ROM, strength, functionality, disability, quality of life (QoL) or daily life activity).<br><br>***References***:<br>⇨ *Dworkin RH, Turk DC, Farrar JT, Haythornthwaite JA, Jensen MP, Katz NP, et al. Core outcome measures for chronic pain clinical trials: IMMPACT recommendations. Pain. 2005;113(1-2):9-19.*<br>⇨ *Taylor AM, Phillips K, Patel KV, Turk DC, Dworkin RH, Beaton D, et al. Assessment of physical function and participation in chronic pain clinical trials: IMMPACT/OMERACT recommendations. Pain. 2016;157(9):1836-50.*
S	Systematic Reviews of RCTs (with or without meta-analysis).<br>Language: Dutch/English<br>Published in the last two decades (Since 2000), in order to retrieve the most recent evidence and find reviews with a decent methodological quality.

Table S2. Search Strategy: PUBMED – Web Of Knowledge - EMBASE
Last update: 02/06/2022

PUBMED<br><br>(PGMs OR "Muscle tightness" OR "Mechanical neck pain" OR "Muscle weakness" OR "Range of motion limitation" OR "Stress injury" OR "Latent myofascial trigger points" OR "Muscle soreness" OR "myofascial pain" OR "chronic myofascial pain" OR "Myofascial triggerpoint*" OR "Myofascial trigger point*" OR triggerpoint OR "trigger point*" OR pain, musculoskeletal[MeSH Terms] OR "musculoskeletal pain*" OR (Pain, Musculoskeletal) OR "Spine"[Mesh Terms] OR "Sacrum"[Mesh Terms] OR "Coccyx"[Mesh Terms] OR "Lumbosacral Region"[Mesh Terms] OR "Back"[Mesh Terms] OR "Neck"[Mesh Terms] OR"Low Back Pain"[Mesh Terms] OR "Sciatica"[Mesh Terms] OR "Neck Pain"[Mesh Terms] OR "low back pain" OR "low backpain" OR "lower back pain" OR "lower backpain" OR LBP OR sciatica OR "neck pain" OR neckpain OR "spinal pain" OR lumbalgia OR "lumbar pain" OR "lumbar back pain" OR "lumbar spine pain" OR "lumbar region pain" OR backache* OR "back ache*" OR "low back ache*" OR "low backache*" OR lumbago OR dorsalgia OR "discogenic pain" OR "thoracic pain" OR "thoracic back pain" OR "upper back pain" OR "cervicalgia" OR "cervical pain" OR (Pains, Musculoskeletal) OR "Back Pains" OR (Pain, Back) OR (Pains, Back) OR Backache OR Backaches OR "Back Ache" OR (Ache, Back) OR (Aches, Back) OR "Back Aches" OR "Back Pain without Radiation" OR "Vertebrogenic Pain Syndrome" OR (Pain Syndrome, Vertebrogenic) OR (Pain Syndromes, Vertebrogenic) OR (Syndrome, Vertebrogenic Pain) OR (Syndromes, Vertebro- genic Pain) OR "Vertebrogenic Pain Syndromes" OR "Back Pain with Radiation" OR "Neck Pains" OR (Pain, Neck) OR (Pains, Neck) OR "Neck Ache*" OR (Ache, Neck) OR (Aches, Neck) OR Cervicalgia* OR Cervicodynia OR Neck- ache* OR "Cervical Pain*" OR (Pain, Cervical) OR (Pains, Cervical) OR "Posterior Cervical Pain*" OR (Cervical Pain, Posterior) OR (Cervical Pains, Posterior) OR (Pain, Posterior Cervical) OR (Pains, Posterior Cervical) OR (Posterior Cervical Pains*) OR (Neck Pain, Posterior) OR (Neck Pains, Posterior) OR (Pain, Posterior Neck) OR (Pains, Poste- rior Neck) OR "Posterior Neck Pain*" OR "Anterior Cervical Pain*" OR (Cervical Pain, Anterior) OR (Cervical Pains, Anterior) OR (Pain, Anterior Cervical) OR (Pains, Anterior Cervical) OR "Anterior Neck Pain*" OR (Neck Pain, An- terior) OR (Neck Pains, Anterior) OR (Pain, Anterior Neck) OR (Pains, Anterior Neck) OR "Musculoskeletal Pain*" OR (Pain, Musculoskeletal) OR (Pains, Musculoskeletal) OR Myalgia OR (Pelvic Girdle Pain)) AND ("dry needling" OR "Deep acupuncture" OR "Intramuscular stimulation" OR "Trigger point acupuncture" OR "Trigger point therapy" OR "insertion of needles") AND (pain[MeSH Terms] OR pain OR range of motion[MeSH Terms] OR "range of motion" OR strength OR daily living activities[MeSH Terms] OR "quality of life" OR disability OR activities of daily living[MeSH Terms] OR "pain relief" OR "reducing pain" OR "brief pain inventory" OR PBI OR "verbal analog scale" OR VAS OR "pain pressure threshold" OR PPT OR "oswestry disability index" OR ODI OR "cervical range of motion" OR "lumbar range of motion" OR "trigger point deactivation" OR "muscle flexibility" OR "musculoskeletal ailments" OR "reduction in pain scores" OR "analgesia")	NUMBER OF ARTICLES = 1165
WEB OF SCIENCE<br><br>TS=((PGMs OR "Muscle tightness" OR "Mechanical neck pain" OR "Muscle weakness" OR "Range of motion limi- tation" OR "Stress injury" OR "Latent myofascial trigger points" OR "Muscle soreness" OR "myofascial pain" OR "chronic myofascial pain" OR "Myofascial triggerpoint*" OR "trigger point*" OR pain, musculoskeletal OR "mus- culoskeletal pain*" OR (Pain, Musculoskeletal) OR "Spine" OR "Sacrum" OR "Coccyx" OR "Lumbosacral Region" OR "Back" OR "Neck" OR"Low Back Pain" OR "Sciatica" OR "Neck Pain" OR "low back pain" OR "low backpain" OR "lower back pain" OR "lower backpain" OR LBP OR sciatica OR "neck pain" OR neckpain OR "spinal pain" OR lumbalgia OR "lumbar pain" OR "lumbar back pain" OR "lumbar spine pain" OR "lumbar region pain" OR back- ache* OR "back ache*" OR "low back ache*" OR "low backache*" OR lumbago OR dorsalgia OR "discogenic pain" OR "thoracic pain" OR "thoracic back pain" OR "upper back pain" OR "cervicalgia" OR "cervical pain" OR (Pains,	NUMBER OF ARTICLES = 691

Musculoskeletal) OR "Back Pains" OR (Pain, Back) OR (Pains, Back) OR Backache OR Backaches OR "Back Ache" OR (Ache, Back) OR (Aches, Back) OR "Back Aches" OR "Back Pain without Radiation" OR "Vertebrogenic Pain Syndrome" OR (Pain Syndrome, Vertebrogenic) OR (Pain Syndromes, Vertebrogenic) OR (Syndrome, Vertebro- genic Pain) OR (Syndromes, Vertebrogenic Pain) OR "Vertebrogenic Pain Syndromes" OR "Back Pain with Radia- tion" OR "Neck Pains" OR (Pain, Neck) OR (Pains, Neck) OR "Neck Ache*" OR (Ache, Neck) OR (Aches, Neck) OR Cervicalgia* OR Cervicodynia OR Neckache* OR "Cervical Pain*" OR (Pain, Cervical) OR (Pains, Cervical) OR "Pos- terior Cervical Pain*" OR (Cervical Pain, Posterior) OR (Cervical Pains, Posterior) OR (Pain, Posterior Cervical) OR (Pains, Posterior Cervical) OR (Posterior Cervical Pains*) OR (Neck Pain, Posterior) OR (Neck Pains, Posterior) OR (Pain, Posterior Neck) OR (Pains, Posterior Neck) OR "Posterior Neck Pain*" OR "Anterior Cervical Pain*" OR (Cervical Pain, Anterior) OR (Cervical Pains, Anterior) OR (Pain, Anterior Cervical) OR (Pains, Anterior Cervical) OR "Anterior Neck Pain*" OR (Neck Pain, Anterior) OR (Neck Pains, Anterior) OR (Pain, Anterior Neck) OR (Pains, Anterior Neck) OR "Musculoskeletal Pain*" OR (Pain, Musculoskeletal) OR (Pains, Musculoskeletal) OR Myalgia OR (Pelvic Girdle Pain)) AND ("dry needling" OR "Deep acupuncture" OR "Intramuscular stimulation" OR "Trigger point acupuncture" OR "Trigger point therapy" OR "insertion of needles") AND (pain OR pain OR range of motion OR "range of motion" OR strength OR daily living activities OR "quality of life" OR disability OR activities of daily living OR "pain relief" OR "reducing pain" OR "brief pain inventory" OR PBI OR "verbal analog scale" OR VAS OR "pain pressure threshold" OR PPT OR "oswestry disability index" OR ODI OR "cervical range of motion" OR "lum- bar range of motion" OR "trigger point deactivation" OR "muscle flexibility" OR "musculoskeletal ailments" OR "reduction in pain scores" OR "analgesia"))	
EMBASE<br><br>(pgms OR 'muscle tightness'/exp OR 'muscle tightness' OR 'mechanical neck pain'/exp OR 'mechanical neck pain' OR 'muscle weakness'/exp OR 'muscle weakness' OR 'range of motion limitation' OR 'stress injury' OR 'latent myofascial trigger points' OR 'muscle soreness'/exp OR 'muscle soreness' OR 'myofascial pain'/exp OR 'myofascial pain' OR 'chronic myofascial pain' OR 'myofascial triggerpoint*' OR 'trigger point*' OR 'musculoskeletal pain'/exp OR 'musculoskeletal pain' OR 'spine'/exp OR 'spine' OR 'sacrum'/exp OR 'sacrum' OR 'coccyx'/exp OR 'coccyx' OR 'lumbosacral region'/exp OR 'lumbosacral region' OR 'back'/exp OR 'back' OR 'neck'/exp OR 'neck' OR 'sciatica' OR 'low back pain'/exp OR 'low back pain' OR 'low backpain'/exp OR 'low backpain' OR 'lower back pain'/exp OR 'lower back pain' OR 'lower backpain' OR lbp OR 'sciatica'/exp OR sciatica OR 'neck pain'/exp OR 'neck pain' OR neckpain OR 'spinal pain'/exp OR 'spinal pain' OR 'lumbalgia'/exp OR lumbalgia OR 'lumbar pain'/exp OR 'lumbar pain' OR 'lumbar back pain' OR 'lumbar spine pain' OR 'lumbar region pain' OR backache* OR 'back ache*' OR 'low back ache*' OR 'low backache*' OR 'lumbago'/exp OR lumbago OR 'dorsalgia'/exp OR dorsalgia OR 'dis- cogenic pain'/exp OR 'discogenic pain' OR 'thoracic pain'/exp OR 'thoracic pain' OR 'thoracic back pain' OR 'upper back pain'/exp OR 'upper back pain' OR 'cervicalgia'/exp OR 'cervicalgia' OR 'cervical pain'/exp OR 'cervical pain' OR 'back pains' OR 'backache'/exp OR backache OR backaches OR 'back ache'/exp OR 'back ache' OR 'back aches' OR 'back pain without radiation' OR 'vertebrogenic pain syndrome' OR 'vertebrogenic pain syndromes' OR 'back pain with radiation' OR 'neck pains' OR 'neck ache*' OR cervicalgia* OR cervicodynia OR neckache* OR 'cervical pain*' OR 'posterior cervical pain*' OR 'posterior neck pain*' OR 'anterior cervical pain*' OR 'anterior neck pain*' OR 'musculoskeletal pain*' OR 'myalgia'/exp OR myalgia) AND ('dry needling'/exp OR 'dry needling' OR 'deep acupuncture' OR 'intramuscular stimulation'/exp OR 'intramuscular stimulation' OR 'trigger point acupuncture' OR 'trigger point therapy'/exp OR 'trigger point therapy' OR 'insertion of needles') AND (((('pain'/exp OR pain OR 'range'/exp OR range) AND of AND ('motion'/exp OR motion) OR 'range of motion'/exp OR 'range of motion' OR 'strength'/exp OR strength OR daily) AND ('living'/exp OR living) AND activities OR 'quality of life'/exp OR 'quality of life' OR 'disability'/exp OR disability OR activities) AND of AND daily AND ('living'/exp OR living) OR 'pain re- lief'/exp OR 'pain relief' OR 'reducing pain' OR 'brief pain inventory'/exp OR 'brief pain inventory' OR 'pbi'/exp OR pbi OR 'verbal analog scale'/exp	NUMBER OF ARTICLES = 428

OR 'verbal analog scale' OR vas OR 'pain pressure threshold'/exp OR 'pain pressure threshold' OR ppt OR 'oswestry disability index'/exp OR 'oswestry disability index' OR odi OR 'cervical range of motion'/exp OR 'cervical range of motion' OR 'lumbar range of motion' OR 'trigger point deactivation' OR 'muscle flexibility'/exp OR 'muscle flexibility' OR 'musculoskeletal ailments' OR 'reduction in pain scores' OR 'analgesia'/exp OR 'analgesia')	
