# Supplementary material for: Clinical Effectiveness of Dry Needling in Patients with Musculoskeletal Pain—An Umbrella Review
Source: J Clin Med. 2023 Feb 2;12(3):1205. doi: 10.3390/jcm12031205 (PMC9917679; doi:10.3390/jcm12031205)
Supplement: Supplementary file 1 [file jcm-12-01205-s001.zip › Suppl. Material_Table S3_Reference list primary studies.pdf]

Table S3. Reference list primary studies

1.	Irnich D, Behrens N, Gleditsch J, Gleditsch J, Schrieber M, Schops P, et al. Immediate effects of dry needling and acupuncture at distant points in chronic neck pain: results of a randomized, double-blind, sham-controlled crossover trial. Pain. 2002;99(1):83–9.
2.	Edwards J, Knowles N. Superficial dry needling and active stretching in the treatment of myofascial pain – a randomised controlled trial. Acupunct Med. 2003;21(3):80–6.
3.	Huguenin L, Brukner PD, McCrory P, Smith P, Wajswelner H, Bennell K. Effect of dry needling of gluteal muscles on straight leg raise: a randomized, placebo controlled, double blind trial. Br J Sports Med. 2005;38(2):84–90.
4.	Itoh K, Saito S, Sahara S, Naitoh Y, Imai K, Kitakoji H. Randomized trial of trigger point acupuncture treatment for chronic shoulder pain: a preliminary study. J Acupunct Meridian Stud. 2014;7(2):59–64.
5.	Ay S, Evcik D, Tur BS. Comparison of injection method in myofascial pain syndrome a randomized controlled trial. J Clin Rheumatol. 2010;29(1):19–23.
6.	Fernandez-Carnero J, La Touche R, Ortega-Santiago R, Galan-del- Rio F, Pesquera J, Ge HY, et al. Short-term effects of dry needling of active myofascial trigger points in the masseter muscle in patients with temporomandibular disorders. J Orofac Pain. 2010;24(1):106–12.
7.	Perez-Palomares S, Olivan-Blazquez B, Magallon-Botaya R, De-la-Torre-Beldarrain MML, Gaspar-Calvo E, Romo-Calvo L, et al. Percutaneous electrical nerve stimulation versus dry needling: effectiveness in the treatment of chronic low back pain. J Musculoskelet Pain. 2010;18(1):23–30.
8.	Srbely JZ, Dickey JP, Lee D, Lowerison M. Dry needle stimulation of myofascial trigger points evokes segmental anti-nociceptive effects. J Rehabil Med. 2010;42(5):463–8.
9.	Diracoglu D, Vural M, Karan A, Aksoy C. Effectiveness of dry needling for the treatment of temporomandibular myofascial pain: a double-blind, randomized, placebo controlled study. J Back Musculoskelet Rehabil. 2012;25(4):285–90.
10.	Eftekhar-Sadat B, Babaei-Ghazani A, Zeinolabedinzadeh V. Evaluation of dry needling in patients with chronic heel pain due to plantar fasciitis. Foot (Edinb). 2012;:1–5.
11.	Myburgh C, Hartvigsen J, Aagaard P, Holsgaard-Larsen A. Skeletal muscle contractility, self-reported pain and tissue sensitivity in females with neck/shoulder pain and upper trapezius myofascial trigger points – a randomized intervention study. Chiropr Man Therap. 2012;20(1):1–10.
12.	Tsai C, Hsieh L, Kuan T, Kao M, Chou L, Hong C. Remote effects of dry needling on the irritability of the myofascial trigger point in the upper trapezius muscle. Am J Phys Med Rehabil. 2010;89(2):133–40.
13.	Eroglu P, Yilmaz O, Bodur H, Ates C. A comparison of the efficacy of dry needling, lidocaine injection, and oral flurbiprofen treatments in patients with myofascial pain syndrome: a double-blind (for injection, groups only), randomized clinical trial. Arch Rheum. 2013;28(1):38– 46.
14.	Mayoral O, Salvat I, Martin M, Martin S, Santiago J, Cotarelo J, et al. Efficacy of myofascial trigger point dry needling in the prevention of pain after total knee arthroplasty: a randomized, double-blinded, placebo-controlled trial. Evid Based Complement Alternat Med. 2013;2013(694941):1–8.
15.	Tekin L, Akarsu S, Durmus O, Cakar E, Dincer U, Kiralp MZ. The effect of dry needling in the treatment of myofascial pain syndrome: a randomized double-blinded placebo-controlled trial. Clin Rheumatol. 2013;32(3):309–15.
16.	Cotchett MP, Munteanu SE, Landorf KB. Effectiveness of trigger point dry needling for plantar heel pain: a randomized controlled trial. Phys Ther. 2014;94:1083–94.
17.	Mejuto-Vazquez MJ, Salom-Moreno J, Ortego-Santiago R, Truyols- Dominguez S, Fernandez-de-Las-Penas C. Short-term changes in neck pain, widespread pressure pain sensitivity, and cervical range of motion after the application of trigger point dry needling in patients with acute mechanical neck pain: a randomized clinical trial. J Orthop Sports Phys Ther. 2014;44(1):252–61.
18.	Zheng Y, Shi D, Wu X, Gu M, Ai Z, Tang K, et al. Ultrasound-guided miniscalpel-needle release versus dry needling for chronic neck pain: a randomized controlled trial. Evid Based Complement Alternat Med. 2014;2014:1–8.
19.	Llamas-Ramos R, Pecos-Martin D, Gallego-Izquierdo T, Llamas- Ramos I, Plaza-Manzano G, Ortego-Santiago R, et al. Comparison of the short-term outcomes between trigger point dry needling and trigger point manual therapy for the management of chronic mechanical neck pain: a randomized clinical trial. J Orthop Sports Phys Ther. 2014;44(11):852–61.

20. Hong, C.Z., Chen, Y.C., Pon, C.H., Yu, J., 1993. Immediate effects of various physical medicine modalities on pain threshold of an active myofascial trigger point. J. Musculoskelet. Pain 1 (2), 37e53.
21. Chu, J., 1997. Does EMG (dry needling) reduce myofascial pain symptoms due to cervical nerve root irritation? Electromyogr. Clin. Neurophysiol. 37, 259e272.
22. Ilbuldu, E., Cakmak, A., Disci, R., Aydin, R., 2004. Comparison of laser, dry needling, and placebo laser treatments in myofascial pain syndrome. Photomed. Laser Ther. 22 (4), 306e311.
23. DiLorenzo, L., Traballesi, M., Morelli, D., Pompa, A., Brunelli, S., Buzzi, M.G., Formisano, R., 2004. Hemiparetic shoulder pain syndrome treated with deep dry needling during early rehabilitation: a prospective, open-label, randomized investigation. J. Musculoskelet. Pain 12 (2), 25e34.
24. Kamanli, A., Kaya, A., Ardicoglu, O., Ozgocmen, S., Zengin, F.O., Bayık, Y., 2005. Comparison of lidocaine injection, botulinum toxin injection, and dry needling to trigger points in myofascial pain syndrome. Rheumatol. Int. 25 (8), 604e611.
25. Itoh, K., Katsumi, Y., Hirota, S., Kitakoji, H., 2007. Randomised trial of trigger point acupuncture compared with other acupuncture for treatment of chronic neck pain. Complement. Ther. Med. 15 (3), 172e179.
26. Ga, H., Choi, J.H., Park, C.H., Yoon, H.J., 2007. Dry needling of trigger points with and without paraspinal needling in myofascial pain syndromes in elderly patients. J. Altern. Complement. Med. 13 (6), 617e624.
27. Hsieh, Y.L., Kao, M.J., Kuan, T.S., Chen, S.M., Chen, J.T., Hong, C.Z., 2007. Dry needling to a key myofascial trigger point may reduce the irritability of satellite MTrPs. Am. J. Phys. Med. Rehabil. 86 (5), 397e403.
28. Casanueva, B., Rivas, P., Rodero, B., Quintial, C., Llorca, J., Gonzalez-Gay, M.A., 2014. Short-term improvement following dry needle stimulation of tender points in fibromyalgia. Rheumatol. Int. 34 (6), 861e866.
29. Santos, R.B.D.C., Carneiro, M.I.S., Oliveira, D.M.D., Maciel, A.B.D.R., Monte- Silva, K.K.D., Araújo, M.D.G.R., 2014. Impact of dry needling and ischemic pressure in the myofascial syndrome: controlled clinical trial. Fisioterapiaem Mov. 27 (4), 515e522.
30. Ziaeifar, M., Arab, A.M., Karimi, N., Nourbakhsh, M.R., 2014. The effect of dry needling on pain, pressure pain threshold and disability in patients with a myofascial trigger point in the upper trapezius muscle. J. Bodyw. Mov. Ther. 18 (2), 298e305.
31. Arias-Buría, J.L., Valero-Alcaide, R., Cleland, J.A., Salom-Moreno, J., Ortega- Santiago, R., Atín-Arratibel, M.A., Fernandez-de-las-Pen~as, C., 2015. Inclusion of trigger point dry needling in a multimodal physical therapy program for postoperative shoulder pain: a randomized clinical trial. J. Manip. Physiol. Therapeut. 38 (3), 179e187.
32. Campa-Moran, I., Rey-Gudin, E., Fernandez-Carnero, J., Paris-Alemany, A., Gil- Martinez, A., Lerma Lara, S., et al., 2015. Comparison of Dry Needling versus Orthopedic Manual Therapy in Patients with Myofascial Chronic Neck Pain: a Single-Blind, Randomized Pilot Study. Pain Research and Treatment.
33. Pecos-Martín, D., Montan~ez-Aguilera, F.J., Gallego-Izquierdo, T., Urraca-Gesto, A., Gomez-Conesa, A., Romero-Franco, N., Plaza-Manzano, G., 2015. Effectiveness of dry needling on the lower trapezius in patients with mechanical neck pain: a randomized controlled trial. Arch. Phys. Med. Rehabil. 96 (5), 775e781.
34. Salom-Moreno, J., Ayuso-Casado, B., Tamaral-Costa, B., Sanchez-Mila, Z., Fernandez de-las- Pen~as, C., Alburquerque-Sendín, F., 2015. Trigger Point Dry Needling and Proprioceptive Exercises for the Management of Chronic Ankle Instability: a Randomized Clinical Trial. Evidence Based Complementary and Alternative Medicine.
35. Garvey TA, Marks MR, Wiesel SW. A prospective, randomized, double-blind evaluation of trigger-point injection therapy for low- back pain. Spine 1989;14:962-4.
36. Hesse J, Mogelvang B, Simonsen H. Acupuncture versus meto- prolol in migraine prophylaxis: a randomized trial of trigger point inactivation. J Intern Med 1994;235:451-6.
37. Hong CZ. Lidocaine injection versus dry needling to myofascial trigger point. The importance of the local twitch response. Am J Phys Med Rehabil 1994;73:256-63.
38. McMillan AS, Nolan A, Kelly PJ. The efficacy of dry needling and procaine in the treatment of myofascial pain in the jaw muscles. J Orofac Pain 1997;11:307-14.

39. Couto C, de Souza IC, Torres IL, Fregni F, Caumo W. Paraspinal stimulation combined with trigger point needling and needle rotation for the treatment of myofascial pain: a randomized sham-controlled clinical trial. Clin J Pain. 2014;30(3):214–223.
40. Sterling M, Vicenzino B, Souvlis T, Con-nelly LB. Dry-needling and exercise for chronic whiplash-associated disorders: a randomized single-blind placebo-controlled trial. Pain. 2015;156:635-643. https://doi.org/10.1097/01.j.p ain.0000460359.40116.c1
41. Ahn SL, Woo JW, Kim JA, et al. A comparison of NSAID and intramuscular stimulation therapy effectiveness in the female patient with chronic shoulder pain. J Korean Geriatr Soc 2002;6:55–66.
42. Gunn CC, Milbrandt WE, Little AS, et al. Dry needling of muscle motor points for chronic low-back pain: a randomized clinical trial with long-term follow-up. Spine 1980;5:279–91.
43. Karakurum B, Karaalin O, Coskun O, et al. The 'dry-needle technique': intramuscular stimulation in tension-type headache. Cephalalgia 2001;21:813–17.
44. Byeon HT, Park SH, Ko MH, et al. Effects of intramuscular stimulation in myofascial pain syndrome of upper trapezius muscle. J Korean Acad Rehabil Med 2003;27:753–6. White A, Cummings TM, Filshie J. An introduction to Western medical acupuncture. Edinburgh: Churchill Livingstone/Elsevier, 2008.
45. Cerezo-Téllez E, Torres Lacomba M, Fuentes- Gallardo I, Mayoral del Moral O, Rodrigo-Medina B, Gutiérrez Ortega C. Dry needling of the trapezius muscle in office workers with neck pain: a randomized clinical trial. J Man Manip Ther. 2016;24:223-232. https://doi.org/10.1179/2 042618615Y.0000000004
46. Cerezo-Téllez E, Torres-Lacomba M, Fuentes-Gallardo I, et al. Effectiveness of dry needling for chronic nonspecific neck pain: a randomized, single-blinded, clinical trial. *Pain*. 2016;157:1905-1917. https://doi.org/10.1097/j. pain.0000000000000591
47. Calvo-Lobo C, Pacheco-da-Costa S, Hita- Herranz E. Efficacy of deep dry needling on latent myofascial trigger points in older adults with nonspecific shoulder pain: a randomized, controlled clinical trial pilot study. *J Geriatr Phys Ther*. 2017;40:63-73. https://doi.org/10.1519/ JPT.0000000000000048
48. Calvo-Lobo C, Pacheco-da-Costa S, Martínez- Martínez J, Rodríguez-Sanz D, Cuesta-Álvaro P, López-López D. Dry needling on the infraspinatus latent and active myofascial trigger points in older adults with nonspecific shoulder pain: a randomized clinical trial. J Geriatr Phys Ther. 2018;41:1-13. https://doi.org/10.1519/ JPT.0000000000000079
49. Hsu CY, Lee KH, Huang HC, Chang ZY, Chen HY, Yang TH. Manipulation therapy relieved pain more rapidly than acupuncture among lateral epicondylalgia (tennis elbow) patients: a randomized controlled trial with 8-week follow-up. Evid Based Complement Alternat Med. 2016;2016:3079247. https://doi. org/10.1155/2016/3079247
50. Fink M, Wolkenstein E, Karst M, Gehrke A. Acupuncture in chronic epicondylitis: a randomized controlled trial. *Rheumatology (Oxford)*. 2002;41:205-209. https://doi. org/10.1093/rheumatology/41.2.205
51. Davidson JH, Vandervoort A, Lessard L, Miller L. The effect of acupuncture versus ultrasound on pain level, grip strength and disability in individuals with lateral epicondylitis: a pilot study. Physiother Can. 2001;53:195-202.
52. Zanin MS, Ronchi JM, Silva TC, Fuzaro AC, Araujo JE. Electromyographic and strength analyses of activation patterns of the wrist flexor muscles after acupuncture. *J Acupunct Meridian Stud*. 2014;7:231-237. https://doi.org/10.1016/j. jams.2014.02.005
53. Fleckenstein J, Niederer D, Auerbach K, et al. No effect of acupuncture in the relief of delayed-onset muscle soreness: results of a randomized controlled trial. Clin J Sport Med. 2016;26:471-477. https://doi.org/10.1097/ JSM.0000000000000259
54. Hübscher M, Vogt L, Bernhörster M, Rosenhagen A, Banzer W. Effects of acupuncture on symptoms and muscle function in delayed-onset muscle soreness. *J Altern Complement Med*. 2008;14:1011-1016. https://doi.org/10.1089/ acm.2008.0173
55. Yao E, Gerritz PK, Henricson E, et al. Randomized controlled trial comparing acupuncture with placebo acupuncture for the treatment of carpal tunnel syndrome. *PM R*. 2012;4:367-373. https:// doi.org/10.1016/j.pmrj.2012.01.008
56. Plaster R, Vieira WB, Alencar FA, Nakano EY, Liebano RE. Immediate effects of electroacupuncture and manual acupuncture on pain, mobility and muscle strength in patients with knee osteoarthritis: a randomised controlled trial. Acupunct Med. 2014;32:236-241. https:// doi.org/10.1136/acupmed-2013-010489

57. Haser C, Stöggl T, Kriner M, et al. Effect of dry needling on thigh muscle strength and hip flexion in elite soccer players. Med Sci Sports Exerc. 2017;49:378-383. https://doi.org/10.1249/ MSS.0000000000001111
58. Devereux F, O'Rourke B, Byrne PJ, Byrne D, Kinsella S. The effects of myofascial trigger point release on the power and force production in the lower limb kinetic chain. *J Strength* Cond Res. In press. https://doi.org/10.1519/ JSC.0000000000002520
59. Huang LP, Zhou S, Ao M, Zhao ML, Zhang LQ, Cao LJ. Unilateral intramuscular needling can improve ankle dorsiflexor strength and muscle activation in both legs. J Exerc Sci Fit. 2015;13:86-93.
60. Huang LP, Zhou S, Lu Z, et al. Bilateral effect of unilateral electroacupuncture on muscle strength. J Altern Complement Med. 2007;13:539-546. https://doi.org/10.1089/acm.2007.6250
61. Zhou S, Huang LP, Liu J, Yu JH, Tian Q, Cao LJ. Bilateral effects of 6 weeks' unilateral acupuncture and electroacupuncture on ankle dorsiflexors muscle strength: a pilot study. Arch Phys Med Rehabil. 2012;93:50-55. https://doi. org/10.1016/j.apmr.2011.08.010
62. Rossi A, Blaustein S, Brown J, et al. Spinal and peripheral dry needling versus peripheral dry needling alone among individuals with a history of lateral ankle sprain: a randomized controlled trial. Int J Sports Phys Ther. 2017;12:1034-1047.
63. García M, Climent JM, Marimon V, Garrido AM, Pastor G, López C. Estudio comparativo de dos técnicas de infiltración miofascial en puntos gatillo: punción seca e inyec- ción de anestésico local. Rehabilitación 2006; 40(4): 188-192.
64. García R, Tormos L, Vilanova P, Morales R, Pérez A, Segu- ra E. Efectividad de la punción seca de un punto gatillo miofascial versus manipulación de codo sobre el dolor y fuerza máxima de prensión de la mano. Fisioterapia 2011; 33(6): 248-255.
65. Venâncio Rde A, Alencar FG, Zamperini C. Different substances and dry-needling injections in patients with myofascial pain and headaches. Cranio 2008; 26(2): 96-103.
66. Bahadir C, Majlesi J, Unalan H. The effect of high-power pain threshold ultrasound therapy on the electrical activity of trigger points and local twitch response on electromyog- raphy: a preliminary study. J Musculoskelet Pain 2009; 17 (2): 162-172.
67. González LM, Infante P, Granados M, Urresti-Lopez FJ. Treatment of temporomandibular myofascial pain with deep dry needling. Med Oral Patol Oral Cir Bucal 2012; 17(5): e781-e785.
68. Zarei H, Bervis S, Piroozi S, Motealleh A. Added value of gluteus Medius and Quadratus Lumborum dry needling in improving knee pain and function in female athletes with Patellofemoral pain syndrome: a randomized clinical trial. Arch Phys Med Rehabil. 2020;101:265–274.
69. Castro-Sánchez AM, Garcia-López H, Fernández-Sánchez M, et al. Benefits of dry needling of myofascial trigger points on auto- nomic function and photoelectric plethysmography in patients with fibromyalgia syndrome. Acupunct Med. 2020.
70. Tabatabaiee A, Takamjani IE, Sarrafzadeh J, Salehi R, Ahmadi M. Ultrasound-guided dry needling decreases pain in patients with piriformis syndrome. Muscle Nerve. 2019;60:558–565.
71. Onat SS, Polat CS, Bicer S, Sahin Z, Tasoglu O. Effect of dry needling injection and kinesiotaping on pain and quality of life in patients with mechanical neck pain. Pain Physician. 2019;22: 583–589.
72. Martín-Rodríguez A, Sáez-Olmo E, Pecos-Martín D, Calvo-Lobo C. Effects of dry needling in the sternocleidomastoid muscle on cervical motor control in patients with neck pain: a randomised clinical trial. Acupunct Med. 2019;37:151–163.
73. Manafnezhad J, Salahzadeh Z, Salimi M, Ghaderi F, Ghojazadeh M. The effects of shock wave and dry needling on active trigger points of upper trapezius muscle in patients with non-specific neck pain: a randomized clinical trial. J Back Musculoskelet Rehabil. 2019;32:811–818.
74. Luan S, Zhu ZM, Ruan JL, et al. Randomized trial on comparison of the efficacy of extracorporeal shock wave therapy and dry needling in myofascial trigger points. Am J Phys Med Rehabil. 2019;98:677–684.
75. Kamali F, Mohamadi M, Fakheri L, Mohammadnejad F. Dry needling versus friction massage to treat tension type headache: a randomized clinical trial. J Bodyw Mov Ther. 2019;23:89–93.
76. Gildir S,Tüzün EH,Erog˘luG,EkerL.Arandomizedtrialoftrigger point dry needling versus sham needling for chronic tension-type headache. Medicine. 2019;98:e14520.
77. Ceballos-Laita L, Jiménez-del-Barrio S, Marín-Zurdo J, et al. Effects of dry needling in HIP muscles in patients with HIP osteoarthritis: a randomized controlled trial. Musculoskelet Sci Pract. 2019;43:76–82.

78. Bagcier F, Yilmaz N. The impact of extracorporeal shock wave therapy and dry needling combination on the pain, grip strength and functionality in patients diagnosed with lateral epicondylitis. Turk J Osteoporos. 2019;25:65–71.
79. Aydın T, Dernek B, Ege TS, Karan A, Aksoy C. The effectiveness of dry needling and exercise therapy in patients with dizziness caused by cervical myofascial pain syndrome; prospective randomized clinical study. Pain Med. 2019;20:153–160.
80. Sánchez-Romero EA, Pecos-Martín D, Calvo-Lobo C, et al. Effects of dry needling in an exercise program for older adults with knee osteoarthritis: a pilot clinical trial. Medicine. 2018;97:e11255–e11255.
81. Rahbar M, Eslamian F, Toopchizadeh V, et al. A comparison of the efficacy of dry-needling and extracorporeal shockwave therapy for plantar fasciitis: a randomized clinical trial. Iran Red Crescent Med J. 2018;20.
82. Gaubeca-Gilarranz A, Fernandez-De-Las-Penasr C, Medina-Torres JR, et al. Effectiveness of dry needling of rectus abdominis trigger points for the treatment of primary dysmenorrhoea: a randomised parallel-group trial. Acupunct Med. 2018;36:302–310.
83. Velázquez-Saornil J, Ruíz-Ruíz B, Rodríguez-Sanz D, et al. Efficacy of quadriceps vastus medialis dry needling in a rehabilitation protocol after surgical reconstruction of complete anterior cruciate ligament rupture. Medicine. 2017;96:e6726.
84. Tu¨zu¨n EH, Gildir S, Angin E, et al. Effectiveness of dry needling versus a classical physiotherapy program in patients with chronic low-back pain: a single-blind, randomized, controlled trial. J Phys Ther Sci. 2017;29:1502–1509.
85. Tas¸og˘lu O¨, S¸ahin Onat S¸, Bo¨lu¨k H, Tas¸og˘lu I˙, O¨zgirgin N. Com- parison of two different dry-needling techniques in the treatment of myofascial pain syndrome. Agri. 2017;29:9–16.
86. Sobhani V, Shamsoddini A, Khatibi-Aghda A, et al. Effectiveness of dry needling, manual therapy, and Kinesio taping® for patients with chronic Myofascial neck pain: a single-blind clinical trial. Trauma Mon. 2017;22:e39261–e39261.
87. Espí-López GV, Serra-Añó P, Vicent-Ferrando J, et al. Effectiveness of inclusion of dry needling in a multimodal therapy program for patellofemoral pain: a randomized parallel-group trial. J Orthop Sports Phys Ther. 2017;47:392–401.
88. Mahmoudzadeh A, Rezaeian ZS, Karimi A, Dommerholt J. The effect of dry needling on the radiating pain in subjects with discogenic low-back pain: a randomized control trial. J Res Med Sci. 2016;21:86.
89. Segura-Ortí E, Prades-Vergara S, Manzaneda-Piña L, Valero- Martínez R, Polo-Traverso JA. Trigger point dry needling versus strain-counterstrain technique for upper trapezius myofascial trig- ger points: a randomised controlled trial. Acupunct Med. 2016;34: 171–177.
90. Arias-Buría JL, Fernández-de-Las-Peñas C, Palacios-Ceña M, Kop- penhaver SL, Salom-Moreno J. Exercises and dry needling for subacromial pain syndrome: a randomized parallel-group trial. J Pain 2017;18:11–18.
91. Pérez-Palomares S, Oliván-Blázquez B, Pérez-Palomares A, et al. Contribution of dry needling to individualized physical therapy treatment of shoulder pain: a randomized clinical trial. J Orthop Sports Phys Ther. 2017;47:11–20.
92. Salom-Moreno J, Jiménez-Gómez L, Gómez-Ahufinger V, et al. Effects of low-load exercise on Postneedling-induced pain after dry needling of active trigger point in individuals with subacromial pain syndrome. PM R. 2017;9:1208–1216.
93. Ziaeifar M, Arab AM, Mosallanezhad Z, Nourbakhsh MR. Dry needling versus trigger point compression of the upper trapezius: a randomized clinical trial with two-week and three-month follow- up. J Man Manip Ther. 2019;27:152–161.
94. Eftekharsadat B, Babaei-Ghazani A, Zeinolabedinzadeh V. Dry needling in patients with chronic heel pain due to plantar fasciitis: A single-blinded randomized clinical trial. Med J Islam Repub Iran. 2016; 30:401.
95. Gu¨ngo¨r E, Karakuzu GZ. Comparison of the efficacy of corticosteroid, dry needling, and PRP application in lateral epicondylitis. Eur J Orthop Surg Traumatol. 2021. https://doi.org/10.1007/s00590-021-03138-2.
96. Rastegar S, Baradaran Mahdavi S, Hoseinzadeh B, et al. Comparison of dry needling and steroid injection in the treatment of plantar fasciitis: a single-blind randomized clinical trial. Int Orthop. 2018;42(1):109–16.

97. Brennan KL, Allen BC, Maldonado YM. Dry needling versus cortisone injection in the treatment of greater trochanteric pain syndrome: a non- inferiority randomized clinical trial. J Orthop Sport Phys. 2017;47(4):232–9.
98. Uygur E, Aktas ̧B, Özkut A, Erinc ̧S, Yilmazoglu EG. Dry needling in lateral epicondylitis: a prospective controlled study. Int Orthop. 2017;41:2321–2325.
99. Uygur E, Aktas ̧B, Eceviz E, et al. Preliminary report on the role of dry needling versus corticosteroid injection, an effective treatment method for plantar fasciitis: a randomized controlled trial. J Foot Ankle Surg. 2019;58(2):301–5.
100. Uygur E, Aktas ̧B, Yilmazoglu EG. The use of dry needling vs. corticoster- oid injection to treat lateral epicondylitis: a prospective, randomized, controlled study. J Shoulder Elbow Surg. 2021;30(1):134–9.
101. Itoh K, Katsumi Y, Kitakoji H. Trigger point acupuncture treatment of chronic low back pain in elderly patients–a blinded RCT. Acupunct Med 2004;22: 170–7.
102. Itoh K, Katsumi Y, Hirota S, Kitakoji H. Effects of trigger point acupuncture on chronic low back pain in elderly patients–a sham-controlled randomised trial. Acupunct Med 2006;24:5–12.
103. A. Tough, A. R. White, S. H. Richards, and J. L. Campbell,"Myofascial trigger point needling for whiplash associated pain - a feasibility study," Manual erapy, vol. 15, no. 6,pp. 529–535, 2010.
104. G. M. Gallego-Sendarrubias, D. Rodr ́ıguez-Sanz, C. Calvo- Lobo, and J. L. Mart ́ın, "Efficacy of dry needling as an adjunct to manual therapy for patients with chronic mechanical neck pain: a randomised clinical trial," Acupuncture in Medicine, vol. 38, 2020.
105. F. Stieven, G. E. Ferreira, M. Wiebusch, F. X. De Ara ́ujo, L. H. T. da Rosa, and M. F. Silva, "No added benefit of combining dry needling with guideline-based physical therapy when managing chronic neck pain: a randomized controlled trial," e Journal of Orthopaedic & Sports Physical Therapy, vol. 50, pp. 1–21, 2020.
106. J. V. Leon-Hernandez, A. Martin-Pintado-Zugasti, L. G. Frutos et al., "Immediate and short-term effects of the combination of dry needling and percutaneous TENS on post- needling soreness in patients with chronic myofascial neck pain," Brazilian Journal of Physical erapy, vol. 20, no. 5, pp. 422–431, 2016.
107. P. Valiente-Castrillo, A. Mart ́ın-Pintado-Zugasti, C. Calvo-Lobo, H. Beltran-Alacreu, and J. Fern ́andez-Carnero, "Effects of pain neuroscience education and dry needling for the management of patients with chronic myofascial neck pain: a randomized clinical trial," Acupuncture in Medicine, vol. 5, 2020
108. Silva ROF, Conti PCR, Arau ̂jo CRP, Silva RS. Evaluation of dry needling and 0.5% lidocaine injection therapies in myofascial pain trigger points in masticatory muscles. Dental Press J Orthod 2012;17:113–8.
109. Venancio RdeA, Alencar FG, Zamperini C. Botulinum toxin, lidocaine, and dry-needling injections in patients with myofascial pain and headaches. Cranio 2009;27:46–53.
110. Uemoto L, Garcia MA, Gouve ̂a CV, Vilella OV, Alfaya TA. Laser therapy and needling in myofascial trigger point deactivation. J Oral Sci 2013;55:175–81.
111. Gonzalez-Perez LM, Infante-Cossio P, Granados-Nunez M, Urresti-Lopez FJ, Lopez-Martos R, Ruiz-Canela-Mendez P. Deep dry needling of trigger points located in the lateral pterygoid muscle: Efficacy and safety of treatment for management of myo- fascial pain and temporomandibular dysfunction. Med Oral Patol Oral Cir Bucal 2015;20:e326–33.
112. Nixdorf DR, Heo G, Major PW. Randomized controlled trial of botulinum toxin A for chronic myogenous orofacial pain. Pain 2002;99:465–73.
113. Von Lindern JJ, Niederhagen B, Berge ́S, Appel T. Type A botulinum toxin in the treatment of chronic facial pain associated with masticatory hyperactivity. J Oral Maxillofac Surg 2003;61:774–8.
114. Guarda-Nardini L, Manfredini D, Salamone M, Salmaso L, Tonello S, Ferronato G. Efficacy of botulinum toxin in treating myofascial pain in bruxers: a controlled placebo pilot study. Cranio 2008;26:126–35
115. Kurtoglu C, Gur OH, Kurkcu M, Sertdemir Y, Guler-Uysal F, Uysal H. Effect of botulinum toxin-A in myofascial pain patients with or without functional disc displacement. J Oral Maxillofac Surg 2008;66: 1644–1651
116. Ernberg M, Hedenberg-Magnusson B, List T, Svensson P. Efficacy of botulinum toxin type A for treatment of persistent myofascial TMD pain: a randomized, controlled, double-blind multicenter study. Pain 2011;152:1988–96.

117. Christidis N, Nilsson A, Kopp S, Ernberg M. Intramuscular injection of granisetron into the masseter muscle increases the pressure pain threshold in healthy participants and patients with localized myalgia. Clin J Pain 2007;23:467–72.
118. Christidis N, Omrani S, Fredriksson L, Gjelset M, Louca S, Hedenberg-Magnusson B,Ernberg M. Repeated tender point injections of granisetron alleviate chronic myofascial pain—a randomized, controlled, double- blinded trial. J Headache Pain 2015;16:104.
119. Castrillon EE, Cairns BE, Ernberg M, Wang K, Sessle BJ, Arendt-Nielsen L, Svensson P. Effect of peripheral NMDA receptor blockade with ketamine on chronic myofascial pain in temporomandibular disorder patients: a randomized, double-blinded, placebo-controlled Trial. J Orofac Pain 2008;22:122–30.
120. Guarda-Nardini L, Stecco A, Stecco C, Masiero S, Manfredini D. Myofascial pain of the jaw muscles: comparison of short-term effectiveness of botulinum toxin injections and fascial manipulation technique. Cranio 2012;30:95–102.
121. De Carli BM, Magro AK, Souza-Silva BN, Matos Fde S, De Carli JP, Paranhos LR, Magro ED. The effect of laser and botulinum toxin in the treatment of myofascial pain and mouth opening: A randomized clinical trial. J Photochem Photobiol B 2016;159:120–3.
122. Itoh K, Asai S, Ohyabu H, Imai K, Kitakoji H. Effects of trigger point acupuncture treatment on temporomandibular disorders: a preliminary randomized clinical trial. J Acupunct Meridian Stud. 2012;5(2):57---62
123. Ga H, Koh HJ, Choi JH, et al: Intramuscular and nerve root stimulation vs lidocaine injection to trigger points in myofascial pain syndrome. J Rehabil Med 2007;39:374Y8
124. Ma C, Wu S, Li G, et al: Comparison of miniscalpel- needle release, acupuncture needling, and stretching exercise to trigger point in myofascial pain syndrome. Clin J Pain 2010;26:251Y7
125. De Meulemeester KE, Castelein B, Coppieters I, et al. Comparing trigger point dry needling a nd manual pressure technique for the management of myofascial neck/shoulder pain: a randomized clinical trial. J Manipulative Physiol Ther. 2017;40(1):11–20.
126. Rayegani, S.M.; Bayat, M.; Bahrami, M.H.; Raeissadat, S.A.; Kargozar, E. Comparison of dry needling and physiotherapy in treatment of myofascial pain syndrome. Clin. Rheumatol. 2014, 33, 859–864.
127. Aridici, R.; Yetisgin, A.; Boyaci, A.; Tutoglu, A.; Bozdogan, E.; Sen Dokumaci, D.; Kilicaslan, N.; Boyaci, N. Comparison of the Efficacy of Dry Needling and High-Power Pain Threshold Ultrasound Therapy with Clinical Status and Sonoelastography in Myofascial Pain Syndrome. Am. J. Phys. Med. Rehabil. 2016, 95,e149–e158.
128. Hayta, E.; Umdu, N.M. A randomized trial to study the comparison of trigger point dry needling versus Kinesio Taping technique in myofascial pain syndrome during a 3-month follow up. Int. J. Physiother. 2016, 3.
129. Ziaeifar, M.; Arab, A.M.; Nourbakhsh, M.R. Clinical effectiveness of dry needling immediately after application on myofascial trigger point in upper trapezius muscle. J. Chiropr. Med. 2016, 15, 252–258
130. Fernández-Carnero, J.; Gilarranz-De-Frutos, L.; León-Hernández, J.V.; Pecos-Martin, D.; Alguacil-Diego, I.; Gallego-Izquierdo, T.; Martín-Pintado-Zugasti, A. Effectiveness of different deep dry needling dosages in the treatment of patients with cervical myofascial pain: A pilot RCT. Am. J. Phys. Med. Rehabil. 2017, 96, 726–733.
131. Dogan, N.; ¸Sengül, I.; Akçay-Yalbuzda ˘ g, ¸S.; Kaya, T. Kinesio taping versus dry needling in the treatment of myofascial pain of the upper trapezius muscle: A randomized, single blind (evaluator), prospective study.J. Back Musculoskelet. Rehabil. 2019, 32, 819–827.
132. Sukareechai, C.; Sukareechai, S. Comparison of radial shockwave and dry needling therapies in the treatment of myofascial pain syndrome. Int. J. Ther. Rehabil. 2019, 26, 1–8.
133. Arias-Buría, J.L.; Monroy-Acevedo, Á.; Fernández-de-las-Peñas, C.; Gallego-Sendarrubias, G.M.;Ortega-Santiago, R.; Plaza-Manzano, G. Effects of dry needling of active trigger points in the scalene muscles in individuals with mechanical neck pain: A randomized clinical trial. Acupunct. Med. 2020.
134. García-de-Miguel, S.; Pecos-Martín, D.; Larroca-Sanz, T.; Sanz-de-Vicente, B.; Garcia-Montes, L.;Fernandez-Matias, R.; Gallego-Izquierdo, T. Short-term effects of PENS versus dry needling in subjectswith unilateral mechanical neck pain and active myofascial trigger points in levator scapulae muscle:A randomized controlled trial. J. Clin. Med. 2020, 9, 1665.

135. Ga H, Choi JH, Park CH, et al. Acupuncture needling versus lidocaine injection of trigger points in myofascial pain syndrome in elderly patients: A randomised trial. Acupunct Med 2007;254):130–6.
136. Ibrahim DA, Abdelrahem HA. Cervical region trigger point Injection with dry needling versus wet needling by lidocaine in geriatric population: A comparative study. Ain-Shams J Anesthesiol 2019;11(1):4–9.
137. Raeissadat SA, Rayegani SM, Sadeghi F, et al. Comparison of ozone and lidocaine injection efficacy vs dry needling in myofascial pain syndrome patients. J Pain Res 2018;11:1273–9.
138. Lee SM, Hwang KS, Han HC, Jeong HS. Clinical study of different effect between trigger point needling and remote acupuncture point needling on tension-type headache [article in Korean]. Journal ofAcupuncture Research. 2001;18:14–20.
139. Patra RC, Gautam AP, Mohanty P. Effectiveness of dry needling on pain and range of motion in patients with cervicogenic headache. International Journal of Innovative Science and Research Technology. 2017;2:466–469.
140. Patra RC, Mohanty P, Gautam AP. Effectiveness of C1-C2 sustained natural apophyseal glide combined with dry needling on pressure point threshold and headache disability in cervicogenic headache. Asian J Pharm Clin Res. 2018;11:171–174
141. Sedighi A, Ansari NN, Naghdi S. Comparison of acute effects of superficial and deep dry needling into trigger points of suboccipital and upper trapezius muscles in patients with cervicogenic headache. J Bodyw Mov Ther. 2017;21:810–814.
142. Togha M, Bahrpeyma F, Jafari M, Nasiri A. A sonographic comparison of the effect of dry needling and ischemic compression on the active trigger point of the sternocleidomastoid muscle associated with cervicogenic headache: a randomized trial. J Back Musculoskelet Rehabil. 2020;33:749–759.
143. de Abreu, Venâncio R, Guedes Pereira Alencar F, Zamperini C. Different substances and dry-needling injections in patients with myofascial pain and headaches. Cranio. 2008;26:96–103.
144. de Abreu Venâncio R, Guedes Pereira Alencar F Jr, Zamperini C. Botulinum toxin, lidocaine, and dry-needling injections in patients with myofascial pain and headaches. Cranio. 2009;27:46–53.
145. Chou LW, Hsieh YL, Kao MJ, Hong CZ. Remote influences of acupuncture on the pain intensity and the amplitude changes of endplate noise in the myofascial trigger point of the upper trapezius muscle. Arch Phys Med Rehabil 2009;90:905-12.
146. Chou LW, Hsieh YL, Chen HS, Hong CZ, Kao MJ, Han TI. Remote therapeutic effectiveness of acupuncture in treating myofascial trigger point of the upper trapezius muscle. Am J Phys MedRehabil 2011;90:1036-49.
147. Krishnan SK, Benzon HT, Siddiqui T, Canlas B. Pain on intramuscular injection of bupivacaine, ropivacaine, with and without dexamethasone. Reg Anesth Pain Med 2000;25:615-9.
148. Arias-Buría, J.L.; Martín-Saborido, C.; Cleland, J.; Koppenhaver, S.L.; Plaza-Manzano, G.; Fernández-de-Las-Peñas, C. Costeffectiveness evaluation of the inclusion of dry needling into an exercise program for subacromial pain syndrome: Evidence from a randomized clinical trial. Pain Med. 2018, 19, 2336–2347.
149. Imani, M.; Abbasi, L.; Taghizadeh, S.; Amiri, M. Comparison of the effect of two different types of dry-needling techniques on subacromial impingement syndrome. J. Bodyw. Mov. Ther. 2020, 25, 35–40.
150. Kheradmandi, A.; Kamali, F.; Ebrahimian, M.; Abbasi, L. Comparison between dry needling plus manual therapy with manual therapy alone on pain and function in overhead athletes with scapular dyskinesia: A randomized clinical trial. J. Bodyw. Mov. Ther. 2020, 26, 339–346.
151. Kamali, F.; Sinaei, E.; Morovati, M. Comparison of upper trapezius and infraspinatus myofascial trigger point therapy by dry needling in overhead athletes with unilateral shoulder impingement syndrome. J. Sport Rehabil. 2019, 28, 243–249.
152. Halle, R.; Crowell, M.; Goss, D. Dry needling and physical therapy versus physical therapy alone following shoulder stabilization repair: A randomized clinical trial. Int. J. Sports Phys. Ther. 2020, 15, 81–102.
153. Ekici, G.; Özcan, ¸S.; Öztürk, B.Y.; Öztürk, B.; Ekici, B. Effects of deep friction massage and dry needling therapy on night pain and shoulder internal rotation in subacromial pain syndrome: 1-year follow up of a randomised controlled trial. Int. J. Ther. Rehabil. 2021, 28, 1–12.

154. Jalilipanah, P.; Okhovatian, F.; Serri, R.A.; Bagban, A.A.; Zamani, S. The effect of dry needling and muscle energy technique

separately and in combination in patients suffering shoulder impingement syndrome and active trigger points of infraspinatus. J.

Bodyw. Mov. Ther. 2020, 26, 94–100. [

155. Koppenhaver, S.; Embry, R.; Ciccarello, J.; Waltrip, J.; Pike, R.; Walker, M.; Fernández-De-Las-Peñas, C.; Croy, T.; Flynn, T. Effects

of dry needling to the symptomatic versus control shoulder in patients with unilateral subacromial pain syndrome. Man. Ther.

2016, 26, 62–69.

156. Mendigutia-Gómez A, Martín-Hernández C, Salom-Moreno J,Fernández-de-las-Penas ˜ C. Effect of dry needling on spasticity, shoulderrange ofmotion, and pressure pain sensitivity in patients with stroke: a crossover study. J Manip Physiol Ther 2016;39(5):348–58.

157. Liu WD, Xue LK, Tian M, Zhang F. Clinical research on a myofascial pain trigger point combining Baihui acupoint therapy of myofascial pain syndrome and living quality analysis. Int J Clin Exp Med 2016;9(2):866–76.

158. García-Gallego R, Tormos-Claramunt L, VilanovaSalcedo P, et al. Efectividad de la punción seca de un punto gatillo miofascial versus manipulación de codo sobre el dolor y fuerza máxima de prensión de la mano. Fisioterapia 2011; 33(6): 248–255.

159. Sukumar S, Sukumar S and Lawrence Mathias S. Effects of static dry needle insertion and trigger point deactivation combined with eccentric exercises in women with unilateral tennis elbow, a single blinded RCT. Glob J Multidiscip Stud 2014; 4 (1): 411–422.

160. Sukumar S, Lawrence M and Subhashchandra R. Early effects of dry needling and low level laser therapy in chronic tennis elbow - an experimental study. Int J Heal Sci Res 2015; 5(1): 187–196

161. Kheradmandi A, Ebrahimian M, Ghafarinejad F, et al. The effect of dry needling of the trigger points of shoulder muscles on pain and grip strength in patients with lateral epicondylitis: a pilot study. J Rehabil Sci Res 2015; 2(3): 58–62.

162. Etminan Z, Razeghi M and Ghafarinejad F. The effect of dry needling of trigger points in forearm's extensor muscles on the grip force, pain and function of athletes with chronic tennis elbow. J Rehabil Sci Res 2019; 6(1): 27–33.

163. Gattie, E.; Cleland, J.A.; Pandya, J.; Snodgrass, S. Dry Needling Adds No Benefit to the Treatment of Neck Pain: A Sham-Controlled Randomized Clinical Trial With 1-Year Follow-up. J. Orthop. Sports Phys. Ther. 2021, 51, 37–45.

164. Leon-Hernandez, J.V.; Calvo-Lobo, C.; Martin-Pinado-Zugasti, A.; Fernandez-Carnero, J.; Beltran-Alacreu, H. Effectiveness of Dry
Needling with Percutaneous Electrical Nerve Stimulation of High Frequency Versus Low Frequency in Patients with Myofascial
Neck Pain. Pain Physician 2021, 24, 135–143.

165. Stieven, F.; Ferreira, G.; de Araújo, F.; Angellos, R.; Silva, M.; da Rosa, L. Immediate Effects of Dry Needling and Myofascial Release on Local and Widespread Pressure Pain Threshold in Individuals With Active Upper Trapezius Trigger Points: A Randomized Clinical Trial. J. Manipulative Physiol. Ther. 2021, 44, 95–102.

166. Goddard G, Karibe H, McNeill C, Villafuerte E. Acupuncture and sham acupuncture reduce muscle pain in myofascial pain patients. Orofac Pain. 2002;16(1):71-76.

167. Shen YF, Goddard G. The short-term effects of acupuncture on myofascial pain patients after clenching. Pain Pract. 2007;7(3):256-264.

168. Shen YF, Younger J, Goddard G, Mackey S. Randomized clinical trial of acupuncture for myofascial pain of the jaw muscles. J Orofac Pain. 2009;23(4):353-359.

169. Zotelli VL, Grillo CM, Gil ML, Wada RS, Sato JE, da Luz Rosario de Sousa M. Acupuncture effect on pain, mouth opening limitation and on the energy meridians in patients with temporomandibular dysfunction: a randomized controlled trial. J Acupunct Meridian Stud. 2017;10(5):351-359.

170. Nitecka-Buchta A, Walczynska-Dragon K, Batko-Kapustecka J, Wieckiewicz M. Comparison between collagen and lidocaine intramuscular injections in terms of their efficiency in decreasing myofascial pain within masseter muscles: a randomized, single-blind controlled trial. Pain Res Manag. 2018;2018:1-10.
171. Nitecka-Buchta A, Walczynska-Dragon K, Kempa WM, Baron S. Platelet-rich plasma intramuscular injections – antinociceptive therapy in myofascial pain within masseter muscles in temporomandibular disorders patients: a pilot study. Front Neurol. 2019;10:250.
172. Lopez-Martos R, Gonzalez-Perez LM, Ruiz-Canela-Mendez P, Urresti-Lopez FJ, Gutierrez-Perez JL, Infante-Cossio P. Randomized, double-blind study comparing percutaneous electrolysis and dry needling for the management of temporomandibular myofascial pain. Med Oral Patol Oral Cir Bucal. 2018;23(4):e454-e462.
173. Kütük SG, Özkan Y, Kütük M, Özdaş T. Comparison of the efficacies of dry needling and Botox methods in the treatment of myofascial pain syndrome affecting the temporomandibular joint. J Craniofac Surg. 2019;30(5):1556-1559.
174. Faria CAdS. Dry Needling in the Management of Myofascial Trigger Points in the Orofacial Area. Estomatologia/Farmacologia e Terapêutica. Port, Portugal: Univesidade Do Porto; 2014. https:// pdfs.seman ticsc holar.org/98c7/ce002 ab971 edca8 d6404 4aa3c ef8fe f7c474.pdf. Accessed March 19, 2020.
175. Fouda A. Comparison between four treatment modalities for active myofascial triggers points. Plast Aesthet Res. 2014;1(1):21-28.
176. Sabatke S, Scola RH, Paiva ES, Kowacs PA. Injection of trigger points in the temporal muscles of patients with myofascial syndrome. Arq Neuropsiquiatr. 2015;73(10):861-866.
177. Jadhao V, Lokhande N, Habbu S, Sewane S, Dongare S, Goyal N. Efficacy of botulinum toxin in treating myofascial pain and occlusal force characteristics of masticatory muscles in bruxism. Indian Journal of Dental Research. 2017;28(5):493.
178. Sánchez ER, Fernández-Carnero J, Calvo-Lobo C, Ochoa VS, Burgos VC, Pecos-Martín D. Is a Combination of Exercise and Dry Needling Effective for Knee OA? Pain Med. 2020; 21:349-63.
179. Al-Boloushi Z, Gómez-Trullén EM, Bellosta-López P, López-Royo MP, Fernández D, Herrero P. Comparing two dry needling interventions for plantar heel pain: a protocol for a randomized controlled trial. JOrthop Surg Res. 2019; 14:31.
180. Mason JS, Crowell M, Dolbeer J, Morris J, Terry A, Koppenhaver S, et al. The effectiveness of dry needling and stretching vs. stretching alone on hamstring flexibility in patients with knee pain: a randomized controlled trial. Int J Sports Phys Ther. 2016; 11:672.
181. Macdonald AJ, Macrae KD, Master BR, et al. Superfi cial acupuncture in the relief of chronic low back pain. Ann Roy Coll Surg. 1983;65:44-46.
182. Jiang GM, Lin MD, Wang LY. Comparative study on effect of acupuncture and lidocaine block for lumbar myofascial pain syndrome [in Chinese]. Zhongguo Zhen Jiu 2013;33:223–6.
183. Wu SF. The Study Of Shallow Needles All Round Thorns Trigger Point Combined With Massage In The Treatment Of Back Myofascial Pain Syndrome [in Chinese]. Guangzhou Univ Chin Med, Guangzhou:2014.
184. Chen Z. Study on the Super Laser Therapy on Trigger Points for Low Back Myofascial Pain Syndrome [in Chinese]. Guangzhou Univ Chin Med, Guangzhou:2014.
185. Kuang JF. A Research on Acupuncture at Trigger Points Treatment for Chronic Low Back Myofascial Pain Syndrome [in Chinese]. Guangzhou Univ Chin Med, Guangzhou:2013.
186. Nong HS. Clinical research of muscular fasciae trigger point combined with meridian point in the treatment of lumbar back myofascial pain syndrome [in Chinese]. Chin J Chin Med 2013;28:1091–2.
187. Fu LJ. Study on the New Method of Myofascial Pain by Acupuncture Stimulation to Ah Sih Points] [in Chinese]. Beijing Univ Chin Med, Beijing:2011.

188. Tellez-Garcia M, de-la-Llave-Rincon AI, Salom-Moreno J, et al. Neuroscience education in addition to trigger point dry needling for the management of patients with mechanical chronic low back pain: a preliminary clinical trial. J Bodyw Mov Ther 2015;19:464–72
189. Hirota S, Itoh K, Katsumi Y. A controlled clinical trial comparing trigger point acupuncture with tender point acupuncture treatments for chronic low back pain-a pilot study on 9 elderly patients [in Japanese]. Zen Nihon Shinkyu Gakkai Zasshi 2006;56:68–75.
190. Itoh K, Katsumi Y. Effect of acupuncture treatment on chronic low back pain with leg pain in aged patients: a controlled trial about short-term effects of trigger point acupuncture [in Japanese]. J Japn Acupuncture Moxibustion Soc 2005;55:530–7.
191. Shen CG, Ding JM. Clinical observation of effectiveness in the treatment of lumbar disc herniation with intramuscular stimulation therapy 30 cases reports [in Chinese]. Zhejiang J Tradit Chin Med 2015;50:676.
192. Yang XC, Zhou YM. Clinical observation of effectiveness in the treatment of myofascial pain syndrome in the lower back with intramuscular stimulation therapy [in Chinese]. Chin J Rural Med Pharm 2010;17:44–5.
193. Long JJ, Zhuang XQ, Tan SS, et al. Clinical observation of needling of myofascial trigger points and acupoints for myofascial pain syndrome in the lower back [in Chinese]. J Guangxi Univ Chin Med 2012;15:17–9.
194. Abyaneh, H.M.; Mosallanezhad, Z.; Mohammadalizade, H.; Bakhshi, E.; Vahedi, G.; Nourbakhsh, M.R. Physiotherapy with and without superficial dry needling affects pain and muscle strength in patients with patellofemoral pain syndrome. Iran. Rehabil. J. 2016, 14, 23–30.
195. Sutlive, T.G.; Golden, A.; King, K.; Morris, W.B.; Morrison, J.E.; Moore, J.H.; Koppenhaver, S. Short-term effects of trigger point dry needling on pain and disability in subjects with patellofemoral pain syndrome. Int. J. Sports Phys. Ther. 2018, 13, 462–473
196. Patel, Z.; Srivastava, A.; Shyam, A.; Sancheti, P. Immediate Effect of Dry Needling Vs Ultrasound on Releasing Trigger Points in Quadriceps in Patients with Patello-Femoral Pain Syndrome on Pain. Int. J. Physiother. Res. 2019, 7, 3287–3294.
197. Itoh, K.; Hirota, S.; Katsumi, Y.; Ochi, H.; Kitakoji, H. Trigger point acupuncture for treatment of knee osteoarthritis—A preliminary RCT for a pragmatic trial. Acupunct. Med. 2008, 26, 17–26.
198. Sanchez Romero, E.A.; Fernandez-Carnero, J.; Calvo-Lobo, C.; Ochoa Saez, V.; Burgos Caballero, V.; Pecos-Martin, D. Is a Combination of Exercise and Dry Needling Effective for Knee OA? Pain Med. 2020, 21, 349–363.
199. da Graca-Tarrago, M., Deitos, A., Brietzke, A.P., Torres, I.L.S., Stefani, L.C., Fregni, F., Caumo, W., 2016. Electrical intramuscular stimulation in osteoarthritis enhances the inhibitory systems in pain processing at cortical and cortical spinal system. Pain Med. 17 (5), 877e891
200. da Graca-Tarrago, M., Lech, M., Angoleri, L.D.M., Santos, D.S., Deitos, A., Brietzke, A.P., Torres, I.L.S., Fregni, F., Caumo, W., 2019. Intramuscular electrical stimulus potentiates motor cortex modulation effects on pain and descending inhibitory systems in knee osteoarthritis: a randomized, factorial, shamcontrolled study. J. Pain Res. 12, 209e221
201. Dunning, J., Butts, R., Young, I., Mourad, F., Galante, V., Bliton, P., Tanner, M., Fernandez-De-Las-Pe nas, C., 2018. Periosteal electrical dry needling as an ~ adjunct to exercise and manual therapy for knee osteoarthritis. Clin. J. Pain 34 (12), 1149e1158.
202. Elbadawy, M.A., 2016. Effectiveness of periosteal stimulation therapy and home exercise program in the rehabilitation of patients with advanced knee osteoarthritis. Clin. J. Pain 1
203. Weiner, D.K., Perera, S., Rudy, T.E., Glick, R.M., Shenoy, S., Delitto, A., 2008. Efficacy of percutaneous electrical nerve stimulation and therapeutic exercise for older adults with chronic low back pain: a randomized controlled trial. Pain 140 (2), 344e357.
204. Weiner, D.K., Moore, C.G., Morone, N.E., Lee, E.S., Kent Kwoh, C., 2013. Efficacy of periosteal stimulation for chronic pain associated with advanced knee osteoarthritis: a randomized, controlled clinical trial. Clin. Therapeut. 35 (11), 1703e1720. h
205. Zhang SP, Yip TP, Li QS. Acupuncture treatment for plantar fasciitis: a randomized controlled trial with six months follow-up. Evid Based Complement Alternat Med. 2011;2011:154108.
206. Li S, Shen T, Liang Y, Zhang Y, Bai B. Miniscalpel-needle versus steroid injection for plantar fasciitis: a randomized controlled trial with a 12-month follow-up. Evid Based Complement Alternat Med. 2014;2014:164714.

207. Kumnerddee W, Pattapong N. Efficacy of electro-acupuncture in chronic plantar fasciitis: a randomized controlled trial. Am J Chin Med. 2012;40(6):1167–1176.
208. Wang L, Guo J, Lin F, et al. Efficacy of warm needling plus Chinese Herb Fumigation in chronic plantar fasciitis: a randomized controlled trial. Modern J Integr Trad Chin West Med. 2016;25:416–417.
209. Qian S, Chen L. Efficacy of warm needling plus Chinese Herb Fumigation in patients with chronic heel pain due to plantar fasciitis: a randomized controlled trial. Shanghai J Acu Mox. 2015;34: 362–363.
210. Bagcier F, Yilmaz N. The impact of extracorporeal shock wave therapy and dry needling combination on pain and functionality in the patients diagnosed with plantar fasciitis. J Foot Ankle Surg 2020;59(4):689–93.
