# Supplementary material for: Clinical Effectiveness of Dry Needling in Patients with Musculoskeletal Pain—An Umbrella Review
Source: J Clin Med. 2023 Feb 2;12(3):1205. doi: 10.3390/jcm12031205 (PMC9917679; doi:10.3390/jcm12031205)
Supplement: Supplementary file 1 [file jcm-12-01205-s001.zip › Suppl. Material_Table S4-S6_CCA_Pairs of Reviews.pdf]

UPPER QUARTER

	Al-Moraissi (2019) (33)	Blanco-Diaz et al. (2022) (34)	Cagnie et al. (2015) (35)	Fernández-De-Las-Peñas. (2021) (36)	Hall et al. (2018) (21)	Kietrys et al. (2013) (37)	Lew et al. (2021) (38)	Liu et al. (2015) (39)	Machado et al. (2018) (40)	Navarro-Santana et al. (2020) (41)	Navarro-Santana et al. (2020) (22)	Navarro-Santana et al. (2021) (42)	Navarro-Santana et al. (2022) (43)	Ong et al. (2013) (44)	Pourahmadi et al. (2021) (45)	Rodríguez-Huguet et al. (2022) (46)
**Blanco-Diaz et al. (2022) (34)**	0,00%															
**Cagnie et al. (2015) (35)**	0,00%	0,00%														
**Fernández-De-Las-Peñas. (2021) (36)**	0,00%	0,00%	0,00%													
**Hall et al. (2018) (21)**	0,00%	5,26%	0,00%	0,00%												
**Kietrys et al. (2013) (37)**	0,00%	0,00%	25,00%	0,00%	9,52%											
**Lew et al. (2021) (38)**	0,00%	0,00%	0,00%	4,00%	6,25%	0,00%										
**Liu et al. (2015) (39)**	0,00%	0,00%	21,74%	3,70%	10,71%	52,38%	4,00%									
**Machado et al. (2018) (40)**	30,00%	0,00%	0,00%	0,00%	0,00%	0,00%	0,00%	0,00%								
**Navarro-Santana et al. (2020) (41)**	0,00%	0,00%	5,88%	2,86%	2,63%	11,11%	21,43%	11,63%	0,00%							
**Navarro-Santana et al. (2020) (22)**	0,00%	0,00%	0,00%	0,00%	0,00%	0,00%	0,00%	0,00%	0,00%	0,00%						
**Navarro-Santana et al. (2021) (42)**	0,00%	15,38%	0,00%	0,00%	13,33%	0,00%	9,09%	0,00%	0,00%	0,00%	0,00%					
**Navarro-Santana et al. (2022) (43)**	0,00%	0,00%	25,00%	0,00%	0,00%	11,76%	0,00%	17,39%	0,00%	0,00%	0,00%	0,00%				
**Ong et al. (2013) (44)**	0,00%	0,00%	0,00%	0,00%	0,00%	30,77%	0,00%	25,00%	0,00%	3,13%	50,00%	0, 00%	0,00%			
**Pourahmadi et al. (2021) (45)**	0,00%	0,00%	18,75%	0,00%	0,00%	0,00%	0,00%	0,00%	0,00%	0,00%	0,00%	0,00%	0,00%	0,00%		
**Rodríguez-Huguet et al. (2022) (46)**	0,00%	0,00%	0,00%	18,75%	0,00%	4,55%	13,33%	3,33%	0,00%	8,33%	0,00%	0,00%	0,00%	0,00%	0,00%	
**Vier et al. (2019) (47)**	12,00%	0,00%	0,00%	0,00%	0,00%	0,00%	0,00%	0,00%	31,58%	0,00%	0,00%	0,00%	0,00%	0,00%	0,00%	0,00%

Table S4: Overlap for pairs of reviews – Upper Quarter.
Overlap thresholds: 0–5% - slight (white), 6–10% - moderate (green), 11–15% - high (yellow), >15% - very high (red).

LOWER QUARTER

	He et al. (2017) (48)	Hu et al.(49)	Khan et al.(50)	Liu et al.(51)	Llurda-Almuzara et al.(52)	Morihisa et al.(53)	Rahou-El-Bachiri et al.(54)
**Hu et al.(49)**	0,00%						
**Khan et al.(50)**	13,33%	0,00%					
**Liu et al.(51)**	0,00%	68,75%	0,00%				
**Llurda-Almuzara et al.(52)**	18,18%	0,00%	14,29%	0,00%			
**Morihisa et al.(53)**	8,33%	4,76%	23,08%	6,25%	9,09%		
**Rahou-El-Bachiri et al.(54)**	0,00%	0,00%	17,65%	0,00%	0,00%	6,67%	
**Ughreja et al.(55)**	0,00%	0,00%	0,00%	0,00%	0,00%	0,00%	11,76%

Table S5: Overlap for pairs of reviews – Lower Quarter.
Overlap thresholds: 0–5% - slight (white), 6–10% - moderate (green), 11–15% - high (yellow), >15% - very high (red).

**WHOLE BODY**	**Boyles et al. (2015) (23)**	**Charles et al.(2019) (24)**	**Cummings et al. (2001) (25)**	**Espejo-Antúnez et al. (2017) (26)**	**Gattie et al. (2017) (27)**	**Kim et al. (2012) (28)**	**Mansfield et al. (2019) (20)**	**Rodríguez-Mansilla et al. (2016) (29)**	**Sánchez-Infante et al. (2011) (30)**	**Sousa Filho et al. (2021) (31)**
**Charles et al. (2019) (24)**	23,53%									
**Cummings et al. (2001) (25)**	0,00%	3,70%								
**Espejo-Antúnez et al. (2017) (26)**	36,00%	46,15%	0,00%							
**Gattie et al. (2017) (27)**	18,52%	44,00%	0,00%	21,74%						
**Kim et al. (2012) (28)**	0,00%	0,00%	0,00%	0,00%	0,00%					
**Mansfield et al. (2019) (20)**	2,56%	4,76%	0,00%	2,86%	6,25%	0,00%				
**Rodríguez-Mansilla et al. (2016) (29)**	26,67%	31,25%	0,00%	21,43%	3,23%	0,00%	2,56%			
**Sánchez-Infante et al. (2011) (30)**	10,91%	18,18%	0,00%	11,76%	19,57%	0,00%	8,62%	3,39%		
**Sousa Filho et al. (2021) (31)**	0,00%	0,00%	0,00%	0,00%	0,00%	0,00%	0,00%	4,17%	0,00%	
**Tough et al. (2009) (32)**	4,00%	15,38%	9,09%	4,76%	0,00%	0,00%	0,00%	13,04%	0,00%	0,00%

Table S6: Overlap for pairs of reviews – Full body.
Overlap thresholds: 0–5% - slight (white), 6–10% - moderate (green), 11–15% - high (yellow), >15% - very high (red).
